# Distinct tau and alpha-synuclein molecular signatures in Alzheimer’s disease with and without Lewy bodies and Parkinson’s disease with dementia

**DOI:** 10.1007/s00401-023-02657-y

**Published:** 2024-01-10

**Authors:** Bram L. van der Gaag, Natasja A. C. Deshayes, John J. P. Breve, John G. J. M. Bol, Allert J. Jonker, Jeroen J. M. Hoozemans, Jean-Philippe Courade, Wilma D. J. van de Berg

**Affiliations:** 1 Section Clinical Neuroanatomy and Biobanking, Department of Anatomy and Neurosciences, Amsterdam UMC, Vrije Universiteit, Amsterdam, The Netherlands; 2 Amsterdam Neuroscience, Program Neurodegeneration, Amsterdam, The Netherlands; 3 Department of Pathology, Amsterdam UMC, Vrije Universiteit, Amsterdam, The Netherlands; 4 Discoveric Bio alpha Ltd., Pfäffikon, Schwyz Switzerland

**Keywords:** Post-translational modifications, Blotting, Immunohistochemistry, Co-pathology

## Abstract

Alpha-synuclein (aSyn) pathology is present in approximately 50% of Alzheimer’s disease (AD) cases at autopsy and might impact the age-of-onset and disease progression in AD. Here, we aimed to determine whether tau and aSyn profiles differ between AD cases with Lewy bodies (AD-LB), pure AD and Parkinson’s disease with dementia (PDD) cases using epitope-, post-translational modification- (PTM) and isoform-specific tau and aSyn antibody panels spanning from the N- to C-terminus. We included the middle temporal gyrus (MTG) and amygdala (AMY) of clinically diagnosed and pathologically confirmed cases and performed dot blotting, western blotting and immunohistochemistry combined with quantitative and morphological analyses. All investigated phospho-tau (pTau) species, except pT181, were upregulated in AD-LB and AD cases compared to PDD and control cases, but no significant differences were observed between AD-LB and AD subjects. In addition, tau antibodies targeting the proline-rich regions and C-terminus showed preferential binding to AD-LB and AD brain homogenates. Antibodies targeting C-terminal aSyn epitopes and pS129 aSyn showed stronger binding to AD-LB and PDD cases compared to AD and control cases. Two pTau species (pS198 and pS396) were specifically detected in the soluble protein fractions of AD-LB and AD subjects, indicative of early involvement of these PTMs in the multimerization process of tau. Other phospho-variants for both tau (pT212/S214, pT231 and pS422) and aSyn (pS129) were only detected in the insoluble protein fraction of AD-LB/AD and AD-LB/PDD cases, respectively. aSyn load was higher in the AMY of AD-LB cases compared to PDD cases, suggesting aggravated aSyn pathology under the presence of AD pathology, while tau load was similar between AD-LB and AD cases. Co-localization of pTau and aSyn could be observed within astrocytes of AD-LB cases within the MTG. These findings highlight a unique pathological signature for AD-LB cases compared to pure AD and PDD cases.

**Supplementary Information:**

The online version contains supplementary material available at 10.1007/s00401-023-02657-y.

## Introduction

Amyloid beta (Aβ)-rich extracellular protein deposits and neuronal inclusions rich in aggregated tau are the neuropathological hallmarks of Alzheimer’s disease (AD). In Parkinson’s disease (PD), inclusion bodies, termed Lewy bodies (LBs) and Lewy neurites (LNs), consisting of damaged organelles, lipids and aggregated alpha-synuclein (aSyn) are the neuropathological hallmarks [59]. However, the presence of tau and aSyn pathology is not mutually exclusive; in approximately 50% of AD cases, aSyn pathology in the brain is common at autopsy and vice versa, in around 50% of PD cases, tau pathology is common [67, 74]. Amygdala (AMY)-predominant aSyn pathology is more commonly observed in early-onset AD than in late-onset AD cases [64]. Moreover, AD cases with LBs (AD-LB) show a more rapid cognitive decline compared to ‘pure’ AD cases [35, 40]. Both proteins show a characteristic and predictable distribution pattern throughout the brain with aging and share common mechanisms in propagation from cell-to-cell [11]. Propagation of pathological tau and aSyn is considered to be conveyed in a prion-like manner; when pathological variants of the proteins propagate to neighboring cells, they act as a template for aggregation of physiological soluble protein species [23]. Tremendous effort is focused on developing disease modifying treatment strategies which are able to slow down or halt disease progression, many of which are focused on combatting propagation of pathological tau and aSyn species via active or passive immunotherapy [13, 16, 44].

In order for therapeutic antibody development to be successful in preventing intercellular propagation of pathological tau and aSyn, it is paramount that these antibodies (1) target epitopes which are available for antibody binding and (2) ideally, target tau or aSyn variants which are elevated in the disease conditions. Specific post-translational modifications (PTMs) have been previously described as being characteristic for aggregated forms of tau and aSyn. Hyperphosphorylation of tau is seen as a driving factor in the pathogenesis of AD and tau pathology has been described to precede Aβ pathology in the brain for roughly a decade, based the current theory that hyperphosphorylation of tau results in its detachment from the microtubules, resulting in aggregation of tau and subsequent formation of neurofibrillary tangles (NFTs), neuronal loss and dysconnectivity [4, 43]. It should be noted however, that phosphorylation is not required per se for driving aggregation of tau into paired helical filaments (PHFs) and kinase inhibitors have thus far shown little effects on clinical outcomes in randomized trials [31, 42]. Alterations in cerebrospinal fluid (CSF) and plasma Aβ42 levels slightly precede changes in total and phospho-tau (pTau) levels, while neuropathological burden of tau pathology correlates better with the severity of cognitive impairment compared to Aβ pathology [28, 50, 71]. Specific phosphorylation sites differ between Braak NFT stages; while some phospho-variants are characteristic for late Braak stages and are enriched in NFTs, others are already involved in the earliest phases of tau multimerization and are seen in earlier Braak stages [19]. Phosphorylation of aSyn at Serine 129 (pS129) is abundant in LBs and LNs in PD(D) patients. Noteworthy, recent studies highlight that this prone PTM is more likely a coping mechanism of the cell to translocate aggregated aSyn from the pre-synapse to the soma for subsequent degradation [3, 6]. Phosphorylation of aSyn at many different sites is common in the soluble protein fraction of brain tissue homogenates, depending on the site and type of modification modulates the interaction with insoluble aSyn aggregates [73]. For both tau and aSyn, many more types of PTMs exist, including glycation, nitration, SUMOylation, acetylation, ubiquitination and truncation [72].

While the relation between Aβ and tau aggregation in the pathogenesis of AD has been thoroughly documented, the interplay between tau and aSyn has been described to a much lesser extent. Co-deposition of tau and aSyn can be observed in neurons in postmortem human brain tissue and recent studies have shown a synergistic relationship between tau and aSyn aggregation [7, 52, 60]. Several studies have highlighted direct interaction between both proteins on a molecular level and have described enhanced propagation and seeding of mixed tau-aSyn aggregates which display distinct configurations [52]. A few recent studies have highlighted interaction between the negatively charged C-terminal domain of aSyn with tau, particularly the positively charged proline-rich region 2 [17, 60].

What is still currently unclear is which variants (epitopes, PTMs and isoforms) of tau and aSyn are characteristic for AD-LB subjects compared to those who display a ‘pure’ pathology. Here, we used a range of tau and aSyn antibodies targeting different epitopes, spanning from the N- to the C-terminal domain, and PTMs to unravel the molecular tau and aSyn signature of AD-LB, AD without LBs (AD) and PD with dementia (PDD) cases and age-matched controls. We included medial temporal gyrus (MTG) and AMY of well-characterized AD-LB, AD, PDD and age-matched control donors (*n* = 10 per group) for dot blotting, western blotting and immunohistochemistry for descriptive and quantitative analyses. A fluorescent multi-labeling staining was performed to assess whether tau and aSyn could co-localize within the same astrocytes. In the current study, we highlight that the molecular signature for tau and aSyn differs between AD-LB cases compared to pure AD and PDD cases.

## Materials and methods

### Study cohort, tissue selection and pathological assessment

For this study, pathology-confirmed postmortem brain tissue from subjects with AD-LB, AD and PDD (*n* = 10 for each of the groups) were acquired from the Netherlands Brain Bank (NBB; Amsterdam, The Netherlands, http://brainbank.nl). Brain tissue from pathologically defined controls (*n* = 10) were obtained from the Normal Aging Brain Collection Amsterdam (NABCA; Amsterdam, The Netherlands, http://nabca.eu). Donors or their next of kin signed informed consent for brain autopsy, the use of brain tissue and the use of medical records for research purposes. The brain donor program of the NBB and NABCA were approved by the local medical ethics committee of the VUmc, Amsterdam (NBB 2009.148; NABCA 2018.150).

Neuropathological diagnosis was established according to international guidelines of Brain Net Europe II (BNE) consortium (http://www.brainnet-europe.org) and NIA-AA criteria for AD [1, 2, 29]. Demographic features and clinical symptoms were retrieved from the clinical files, including sex, age at symptom onset, age at death, disease duration, presence of dementia and parkinsonism, core and supportive clinical features for AD and PD [45, 54].

For pathological diagnosis, 6 µm thin formalin-fixed and paraffin embedded (FFPE) sections were stained using antibodies against Aβ (clone 4G8, Biolegend, 1:8000 dilution), phosphorylated tau (clone AT8, ThermoFisher Scientific, 1:500 dilution) and αSyn (clone KM51, Monosan Xtra, 1:500), as previously described [47]. Braak and McKeith αSyn stages were determined using the BrainNet Europe (BNE) criteria [2]. Based on Thal amyloid-β phases scored on the medial temporal lobe, Braak neurofibrillary stages and CERAD neuritic plaque scores, levels of AD pathology were determined according to on NIA-AA consensus criteria [1, 26, 46, 66]. In addition, Thal CAA stages, presence of aging-related tau astrogliopathy (ARTAG) [34, 65], microvascular lesions, hippocampal sclerosis and APOE genotype were assessed. A summary of the clinical and pathological characteristics for all cases can be found in the supplementary file (Supplementary Table 1).

The regions of interest for this study were the MTG and the AMY, considering the MTG to be a highly vulnerable brain region early in the development of AD and the AMY being a region in which aggregation of aSyn is frequently seen in AD. For both regions, FFPE and frozen tissues were included; FFPE tissue was from the right hemisphere, whereas frozen tissue came from the left hemisphere. Presence of pathological tau and aSyn in the frozen tissue blocks was confirmed by immunostaining (see the Supplementary File).

### Antibody selection, brain tissue homogenization and dot blotting

Antibody panels consisting of commercially available antibodies spanning almost the full amino acid sequence of both tau and aSyn were selected to define the molecular signature of tau and aSyn in the human brain using dot blot (DB), western blot (WB) and immunohistochemistry (IHC) (Supplementary Table 2). All selected antibodies were monoclonal and epitope, isoform or PTM-specific and were previously described in the literature or by the manufacturer to be immunoreactive to human tau or aSyn [19, 22].

Approximately 50–80 mg of frozen tissue was thawed on ice for 10 min after which 500 µL of ice-cold detergent-free lysis buffer was added containing 5 mM HEPES, 320 mM sucrose, 1 × cOmplete mini EDTA-free protease inhibitor cocktail (Roche, cat # 11836170001) and 1 × PhosSTOP phosphatase inhibitor cocktail (Roche, cat# PHOSS-RO) with pH adjusted to 8.0. A 5 mm steel bead was added to each tube and mechanical homogenization was subsequently performed by operating the TissueLyser LT (Qiagen, cat# 85600) for 1 min at 50 Hz. Homogenates were spun down at 1000 × g for 2 min at 4 ℃ and mechanical homogenization was repeated as described above. Homogenates were kept on ice between homogenization steps. To remove endogenous immunoglobulins, 50 µL of settled immobilized Pierce™ Protein A/G (ThermoScientific, cat#20421) was pipetted into separate tubes, after which 500 µL lysis buffer was added and subsequently spun down at 1000 × g for 2 min at 4 ℃. Tissue homogenates were then transferred to the washed sepharose beads and incubated for 2 h at 4 ℃ with gentle mixing. After incubation, tubes were centrifuged at 1000 × g for 10 min at 4 ℃ and the supernatant (sample) was transferred to a new tube. DTT was added to a final concentration of 1 mM to protect samples against oxidation. Protein concentration was determined by BCA (ThermoScientific, cat# 23225) according to manufacturer’s instructions and aliquots were made which were flash frozen on dry ice and subsequently stored at −80 ℃ until further use in the DB assay.

A DB assay was performed to assess antibody binding to crude human brain tissue homogenates as previously described [12]. In short, 1 µg protein per sample was diluted to a total volume of 100 µL TBS for loading on each spot. As a positive control, recombinant protein solutions were prepared; 5 ng recombinant aSyn (rPeptide, cat# S-1001-1) and 5 ng pS129 aSyn (Proteos, cat# RP-004) for the aSyn DBs and 15 ng recombinant tau for all six tau isoforms (TAU441, rPeptide, cat# T1001-1; TAU410, rPeptide, cat# T1002-1; TAU412, rPeptide, cat# T1003-1; TAU381, rPeptide, cat# T1004-1; TAU383, rPeptide, cat# T1005-1; TAU352, rPeptide, cat# T1006-1). All wells without any samples were loaded with TBS as a negative control. Nitrocellulose membranes (LICOR, cat# 1620112) with a pore size of 0.2 µm were cut to the appropriate size to fit the Bio-Dot Apparatus (Bio-Rad, cat# 1706545). Loading of samples and handling of the Bio-Dot Apparatus was performed according to the manufacturer’s instructions. After 1 h, any remaining samples was pulled through the membrane, after which membranes were removed from the apparatus and left to dry on a clean piece of laboratory paper in the fume hood for a minimum duration of 1 h. Membranes were rehydrated in TBS and to determine equal protein loading, Revert™ 700 Total Protein Stain Kit (Licor, cat# 926-11016) was performed according to the manufacturer’s instructions. Imaging was performed after total protein staining on a Licor Odyssey SA imager with the following settings: 100 µm resolution, intensity level 6 and 3.0 mm offset. After imaging, membranes were blocked in Intercept^®^ (TBS) Blocking Buffer (Licor, cat# 927-600001) at RT for 1 h with gentle rocking. After blocking, membranes were incubated overnight in the primary antibody solutions at 4 ℃ (see Supplementary Table 2 for an overview of the selected primary antibody solutions). Following overnight incubation, membranes were washed for 3 × 10 min in TBS-0.1% Tween (TBS-T) at RT. Secondary antibody incubation was then performed with either goat anti-rabbit IRDye^®^ 800CW (Licor, cat# 926-32211, 1:10,000 dilution) or donkey anti-mouse IRDye^®^ 800CW (Licor, cat# 926-32212, 1:10,000 dilution) depending on the host of the primary antibody for 1 h at RT. After secondary antibody incubation, membranes were washed for 2 × 10 min in TBS-T followed by a 1 × 15 min wash in TBS. Membranes were then imaged on a Licor Odyssey SA imager with the same settings as described above.

### Biochemical fractionation, gel electrophoresis and western blotting

Antibodies which showed good performance in the DB experiments in terms of binding and detected epitopes/PTMs which were characteristic for any of the investigated conditions were selected for WB and IHC. Affinity and specificity of these tau and aSyn antibodies (*n* = *6* for both) was evaluated by generating soluble and insoluble protein fractions using differential ultracentrifugation for *n* = *3* cases per investigated condition using an adapted method from an earlier study [18]. In short, 50–80 mg of frozen tissue was mechanically homogenized as mentioned above on the TissueLyser LT with the same settings in 1 mL OG-RIPA buffer containing 1× RIPA (Cell Signaling Technology, cat# 9806S), 1 mM PMSF and 2% octyl glucoside (Avanti, cat# 850511P) [32]. Homogenization and spinning down was repeated for a total of four times after which homogenates were spun down at 1000 × g for 10 min at 4 ℃ to remove any cellular debris. The supernatant was subsequently transferred to ultracentrifuge tubes (Beckman Coulter, cat# 343778) and tubes were balanced. Samples were then centrifuged in the Optima™ MAX-XP Ultracentrifuge (Beckman Coulter, cat # 393315) at 100,000 × g for 1 h at 4 ℃. The supernatant was labeled as the soluble protein fraction, subsequently divided into aliquots, snap frozen on dry ice and stored at −80 ℃ until further use. Pellets were washed with 200 µL OG-RIPA buffer and centrifuged at 100,000 × g for 30 min, after which the supernatant was discarded. 500 µL SDS/U buffer containing 8 M Urea, 5%SDS and TBS was then added to the ultracentrifuge tubes and pellets were sonicated until pellet was dissolved (3/7 setting, 30% amplitude, approximately 10 pulses). Hereafter, suspensions were transferred to new tubes and boiled for 10 min at 100 ℃. This fraction was termed the insoluble protein fraction and was kept at RT. Protein concentration of the samples was determined using a BCA assay according to manufacturer’s instructions.

Gel electrophoresis was performed under reducing conditions according to manufacturer’s instructions. In short, 5 µg protein per sample was loaded into each well. As a positive control for aSyn gels, 20 ng of recombinant aSyn (rPeptide, cat# S1-1001) and 20 ng of recombinant pS129 aSyn (Proteos, cat# RP-004) were loaded per gel, and for tau gels, 2 µL of tau protein ladder (rPeptide, cat# T-1007, containing 5 ng of each tau isoform) was loaded per gel. Samples were prepared in Bolt™ LDS Sample Loading Buffer (Invitrogen, cat# B0007), Bolt™ Sample Reducing Agent (Invitrogen, cat# B0009) and ultrapure water as per manufacturer’s recommendation. After heating the samples for 10 min at 70 ℃, samples were loaded onto Bolt™ Bis–Tris 4–12% gradient gels (Invitrogen, cat# NW04125) and proteins were separated by running gels at 200 V for 35 min with Bolt™ MES-SDS running buffer (Invitrogen, cat# B0002). Bolt™ Antioxidant (Invitrogen, cat# B0005) was added to buffer going into the inner chamber to keep proteins under reduced conditions as recommended per manufacturer. After gel electrophoresis, gels were cut to the appropriate size and equilibrated in 20% ethanol for 5–10 min to aid in protein transfer during blotting.

Dry blotting was performed by placing gels on iBlot 2 Nitrocellulose Transfer Stacks with 0.2 µm pore size (ThermoFisher, cat# IB23001) and running the P0 default setting on the iBlot 2 Gel Transfer Device (ThermoFisher, cat# IB21001). Transfer occurred for a total of 7 min under varying voltages (20–25 V). After transfer, membranes which would be stained for tau were placed on a piece of clean laboratory for 30 min paper to air dry the membrane, while membranes that would be stained for aSyn were fixed in 4% buffered formaldehyde + 0.01% glutaraldehyde for 30 min at RT to improve aSyn detection [57]. Membranes were then rehydrated/washed in TBS for 5 min. Total protein stain was performed and membranes were scanned on the Licor Odyssey SA imager with the same settings described earlier. To allow multiplexing of the membrane, total protein stain was removed as recommended per manufacturer’s instructions, and membranes were scanned once more to assess residual total protein stain. Membranes were subsequently blocked for 1 h at RT using Intercept^®^ (TBS) blocking buffer. After blocking, membranes were multiplexed with two primary antibodies raised in different species and incubation took place overnight at 4 ℃ (same concentration was used as for DB). Multiplex combinations can be found in the supplementary file (Supplementary Table 3). After washing (same as for DB), secondary antibody incubation with donkey anti-mouse IRDye^®^ 680LT (Licor, cat# 926-68022, concentration 1:20,000) and goat anti-rabbit IRDye^®^ 800CW (concentration 1:20,000) was performed for 1 h at RT. Washing followed (same as for DB) after which membranes were imaged with the Licor Odyssey SA imager with the same settings mentioned earlier.

### Immunohistochemistry for descriptive and quantitative analyses

Consecutive FFPE 6 μm-thick tissue sections from MTG and AMY were first deparaffinized using xylene and rehydrated in a series of ethanol with decreasing alcohol percentages. Sections were then subjected to antigen retrieval, which method differed per antibody since our pilot experiments showed different optimal antigen retrieval methods per antibody (see Supplementary Table 4). After antigen retrieval, sections were washed once in TBS and incubated in TBS with 1% H_2_O_2_ or 0.3% H_2_O_2_ + 1% NaN_3_ (only for AT8) for 30 min at RT to block endogenous peroxidase activity. Sections were then washed 3 × 5 min with TBS at RT. To prevent non-specific binding of the primary antibody, sections were blocked in TBS with 1–3% of normal horse serum (NHS) or normal goat serum (NGS) for 30 min at RT. Subsequently, sections were incubated with respective primary antibodies (Supplementary Table 4) diluted in TBS with 0.1% Triton X-100 and 3% NHS or 1–3% NGS overnight at 4 °C. The following day, sections were washed 3 × 5 min with TBS at RT. Primary antibodies were detected using EnVision and visualized using DAB as chromogen. Nuclear staining was then performed using hematoxylin for 60–90 s, after which the sections were dehydrated and mounted using Entellan.

Stained sections were scanned using a whole-slide scanner (Olympus VS200, UPLXAPO 20×/0.80 objective) and regions of interests (ROIs) were subsequently quantified using optimized in-house scripts in QuPath 0.2.3 (https://qupath.readthedocs.io/en/0.2/) [8]. Tissue sections for which the staining or scanning procedure failed were excluded for further analyses. For the MTG, several ROIs in the grey matter and consisting of all cortical layers were outlined. For the AMY, the whole AMY was outlined. Using the QuPath scripts, the load of total tau and aSyn immunoreactivity (%area) and the number of LBs per mm^2^ (for aSyn immunostainings only) were measured. Total tau load included any potential ARTAG present in the section. Images of representative sections were captured by ta Leica DM5000 microscope using the HC PL APO 40×/1.30 oil or HC PL APO 63×/1.40–0.60 oil objective.

### Fluorescent multi-labeling staining to assess co-localization of phospho-tau and aSyn in astrocytes

To determine whether Tau and aSyn could be co-aggregating within the same astrocytes in AD-LB cases, we stained for pSer422 Tau (clone EPR2866, Abcam, cat# ab79415, dilution 1:200), NAC-region aSyn (clone A15115A, BioLegend, cat# 848302, dilution 1:2000) and GFAP (polyclonal, Merck Millipore, cat# AB5541, dilution 1:500) to visualize astrocytes. Deparaffinization of FFPE sections (MTG and AMY) was performed as described before. Antigen retrieval was performed by incubating slides for 10 min in 100% formic acid, after which slides were rinsed under running tap water for 10 min. A second antigen retrieval step was performed by steaming the sections for 10 min in 10 mM Tris–EDTA pH 9.0 buffer. After sections were cooled to room temperature, sections were washed for 5 min in TBS. Blocking was performed for 30 min in 3% normal donkey serum in TBS with 0.01% Triton X-100 (TBS-T). After blocking, primary antibody incubation took place overnight at 4 ℃. The following day, the sections were washed for 3 × 5 min in TBS. To visualize the target proteins, secondary antibody staining was performed at room temperature for 2 h with the following antibodies: donkey anti-chicken Alexa680 (Jackson, cat# 703-625-155, dilution 1:500), goat anti-mouse Star580 (Abberior, cat# ST580-1001, dilution 1:200) and donkey anti-rabbit Alexa488 (Invitrogen, cat# A21206, dilution 1:400). DAPI was used to stain the nuclei. Sections were then washed for 3 × 5 min in TBS, after which slides were cover slipped using Mowiol + DABCO. Confocal laser scanning microscopy (CLSM) was performed on a Leica TCS SP8 and scans were made with a HC PL APO CS2 63×/1.40 NA oil objective. Fluorophores were excited at appropriate wavelengths and hybrid gating (counting mode) was used for detection. Eight sequential Z-scans were made with an overall stack size of 2.1 µm (resolution of 1024 × 1024 pixels) in the MTG. Confocal Z-stack images were corrected for photobleaching and deconvoluted using Huygens Professional Version 23 (Scientific Volume Imaging; Huygens; The Netherlands; https://svi.nl/Huygens-Professional). Fiji (https://imageJ.nih.gov/ij/) was used to display maximum projections. Adobe Illustrator 2023 was used to make the figures.

### Data analysis and statistics

Raw data were processed in Microsoft Excel and statistics were performed using Prism Graphpad (version 9.3.1). Differences for age at onset, age at death, disease duration and PMD between groups were compared with a one-way ANOVA (2-tailed) with Tukey’s multiple comparison’s test. Quantification of blots was represented as normalized values (NVs) to determine the amount of protein of interest detected relative to total protein signal. Exact formulas which were used to determine NVs can be found in the supplementary file. To assess whether biochemical measurements of pathological tau and aSyn were reflective of semi-quantitative manual scoring of pathology in frozen sections from the identical tissue block, Spearman correlations were performed (Supplementary Fig. S2). Distribution of data was evaluated by visual inspection of QQ plots and performing Shapiro–Wilks normality test. If data were normally distributed, a one-way ANOVA (2-tailed) with Tukey’s multiple comparisons test was performed. If data did not fit a Gaussian distribution, the Kruskal–Wallis with Dunn’s correction for multiple comparisons test was performed. Comparisons within and between groups for NVs for the soluble and insoluble protein fractions were made by performing a two-way ANOVA with Tukey’s multiple comparison test (only significant differences between groups within fraction are displayed in graphs). For all statistical tests, *p* < 0.05 was considered significant. Graphs were created in Prism Graphpad and mean ± 95% CI is displayed for all graphs. To visualize the DB data, a clustered heatmap was made in Microsoft Excel.

## Results

### Description of cohort

We included age-matched control donors with little to no pathology (controls; *n* = 10), ‘pure’ AD and PDD cases with little to no concomitant pathologies (*n* = 10 for both) and AD patients with aSyn pathology (AD-LB; *n* = 10). The PDD group had relatively more males than the other groups. However, there were no significant differences in age at death ([68–90 years], *p* = 0.45), age of onset ([53–82 years], *p* = 0.32) and disease duration (*p* = 0.36) between the groups. The AD-LB cases had strong tau and aSyn pathology (Table 1). In contrast, all AD cases had no aSyn pathology (Table 1), while PDD cases had limited AD pathology (Table 1). Control subjects had no aSyn pathology (as evidenced by a Braak aSyn stage of 0 for all cases) and limited AD pathology (Table 1). The PMD was significantly longer for controls compared to the AD-LB (*p* = 0.009) and the PDD group (*p* = 0.02), while CERAD Neuritic score of one PDD donor was missing (Table 1). The presence of tau and aSyn pathology in the frozen tissue blocks was confirmed by immunostaining and manual pathological scoring (Supplementary Fig. S1).

**Table 1:** Clinicopathological characteristics of the donors included in this study

	Control	AD	AD-LB	PDD
Number	10	10	10	10
Sex (M/F)	5/5	6/4	6/4	8/2
Age at death (years ± SD)	76 ± 6	80 ± 8	80 ± 6	77 ± 9
Age at onset (years ± SD)	n.a	70 ± 9	72 ± 6	66 ± 9
Disease duration (years ± SD)	n.a	10 ± 3	9 ± 3	12 ± 6
PMD (h ± min SD)	9 ± 121^*^	7 ± 162	5 ± 69	6 ± 113
Braak LB stage (range and distribution)	0	0	IV–VI (1/0/9)	V–VI (2/8)
Braak NFT stage (range and distribution)	0–II (1/6/3)	IV–VI (3/4/3)	IV–VI (3/3/4)	0–III (1/3/3/3)
Thal amyloid phase (range and distribution)	0–2 (1/5/4)	3–5 (1/2/7)	3–5 (2/4/4)	0–3 (2/6/1/1)
CERAD Neuritic score (range and distribution)	0	1–3 (1/2/7)	0–3 (1/0/2/6)^a^	0

*CERAD* Consortium to Establish a Registry for Alzheimer’s Disease, *M* male, *F* female, *LB* Lewy body, *NFT* neurofibrillary tangles, *PDD* Parkinson’s disease with dementia, *AD* Alzheimer’s disease, *AD-LB* Alzheimer’s disease with aSyn pathology, *PMD* postmortem delay, *SD* standard deviation, *n.a.* not applicable

^*^p < 0.05; PMD was significantly longer in the control compared to the AD-LB and PDD groups

^a^CERAD Neuritic score was missing of one AD-LB case

### Phospho, proline-rich regions and C-terminal tau antibodies display preferential binding to AD-LB and AD cases

**Fig. 1:**
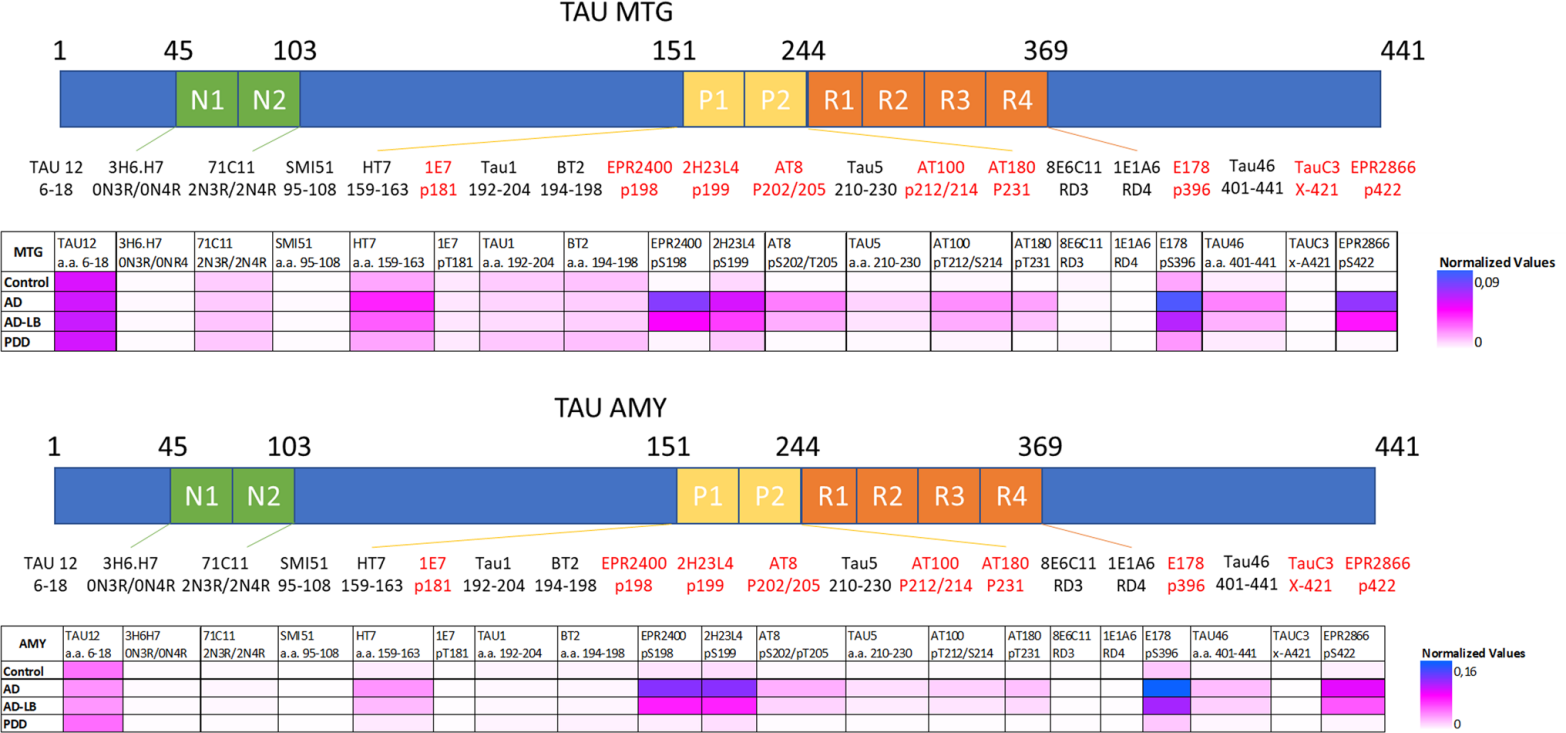
Heatmaps depicting epitope mapping of tau in the human brain. TAU12 (a.a. 6–18) showed best overall binding to both the middle temporal gyrus (MTG) and amygdala (AMY) brain tissues. Phosphorylated tau species were all elevated (except pT181) in both the MTG and AMY of AD and AD-LB cases, with E178 (pS396) being the most abundant phospho-epitope in these groups. Antibodies HT7 (targeting the P1 domain), TAU5 (targeting the P2 domain) and TAU46 (targeting the end of the C-terminus) also showed increased detection of tau in the AD and AD-LB groups. Each tile displays the averaged normalized value per group, as is depicted by the scale bar. PTM-specific antibodies are highlighted in red. *N1/2* N-terminal domain 1 and 2, *P1/2* proline-rich domains 1 and 2, *R1/2/3/4* four microtubule-binding domains (MTBRs), *p* phosphorylation site, *X-* truncation site, *a.a.* amino acid

We tested the binding of 20 tau antibodies (Fig. 1) targeting epitopes spanning the N- to C-terminal domain to crude homogenates of the MTG and AMY tissue from AD-LB, AD, PDD and control cases. TAU12, targeting the N-terminal domain showed the best binding to MTG tissue homogenates from all groups overall (Fig. 1). Some tau antibodies showed little to no binding to MTG tissue homogenates from all cases suggesting an absence of epitope availability or PTM presence: 3H6.H7 (targeting 1N), SMI51 (95–108), 8E6C11 and 1E1A6 (RD3&4), 1E7 (pT181) and Tau C3 (truncated tau 421). Other antibodies such as 71C11 (targeting N2) and BT2 (targeting the PRR 194–198) bound similarly across all groups.

In contrast to Tau1 (targeting the PRR 192–204) and BT2, the other PRR 1 (P1) targeting antibody HT7 (159–163) showed stronger binding to AD-LB brains compared to controls (*p* = 0.04) and PDD (*p* = 0.03) cases (Supplementary Fig. S3). In addition, HT7 also showed stronger binding to AD homogenates compared to controls (*p* < 0.01) and PDD (*p* < 0.01) cases (Supplementary Fig. S3). Similarly, antibody TAU5, targeting the PRR 2 (P2), showed stronger binding to AD-LB cases compared to PDD (*p* = 0.03) and controls (*p* = 0.03) cases (Supplementary Fig. S3). This was also the case when comparing AD cases to controls (*p* < 0.001) and PDD (*p* = 0.03) cases.

Among the pTau-specific antibodies, only 1E7 targeting pT181 showed low signal across groups (Fig. 1). Other p-tau antibodies labelled AD-LB and AD cases more strongly than PDD and control cases. EPR2400 (pS198) showed stronger binding to AD-LB and AD cases compared to controls (*p* < 0.01 and *p* < 0.001, respectively) and PDD (*p* = 0.03 and *p* < 0.001, respectively) cases (Supplementary Fig. S3). 2H23L4 (pS199) showed stronger binding to AD-LB cases compared to controls (*p* < 0.01) but not compared to PDD (*p* > 0.05) cases, while showing stronger binding in AD cases compared to both controls (*p* < 0.0001) and PDD (*p* < 0.01) cases (Supplementary Fig. S3). AT8 (pS202/pT205), AT100 (pT212/S214), AT180 (pT231) and EPR2866 (pS422) showed almost identical results with increased binding to AD-LB and AD MTG tissues compared PDD (*p* < 0.01 and *p* < 0.001, respectively) and to control (*p* = 0.01 and *p* < 0.001, respectively) cases (Supplementary Fig. S3). The C-terminus, phospho-specific E178 (pS396) antibody revealed elevated binding in AD-LB cases compared to controls (*p* = 0.01) but not to PDD (*p* > 0.05) cases, while showing stronger binding to AD cases compared to both controls (*p* < 0.001) and PDD (*p* < 0.01) cases (Supplementary Fig. S3). TAU46, targeting the last 40 amino acids of the C-terminus, also showed elevated binding in AD-LB and AD cases compared to PDD (*p* = 0.04 and *p* < 0.0001, respectively) and control (*p* = 0.03 and *p* < 0.0001, respectively) cases, (Supplementary Fig. S3).

Most antibodies showed a similar binding profile for the AMY (Fig. 1), although some differences were identified when compared to the MTG. For example, TAU12 showed less binding to AD-LB and AD cases compared to controls (*p* < 0.01 and *p* = 0.021, respectively) and compared to PDD (*p* < 0.01 for both) cases (Supplementary Fig. S4). All group differences reported above for the MTG were otherwise similar for the AMY. 2H23L4 and E178 also showed stronger binding in AD-LB cases compared to PDD (*p* = 0.019 and *p* = 0.011, respectively) cases (Supplementary Fig. S4).

In spite of a high variability within groups for the tested tau antibodies (Supplementary Figs. S3, S4), we observed a significant upregulation of phosphorylated tau species (apart from pT181), as well as enrichment of some P1/2 regions and C-terminal epitopes in both the MTG and AMY crude tissue homogenates in AD cases with and without concomitant aSyn pathology when compared to PDD and control cases. Although the beginning of the N-terminus (TAU12) was generally the most available epitope for binding to human brain tissue, pS396 tau (E178) was the most abundant phospho-epitope detected in AD cases with and without concomitant aSyn pathology, closely followed by pS198 (EPR2400), pS199 (2H23L4) and pS422 (EPR2866). These pTau antibodies revealed the most significant group differences when comparing AD-LB and pure AD cases to PDD and control cases. Manual pathological scoring of AT8 in cryosections showed significant correlations with AT8 values as measured with DB (see Supplementary Fig. S2).

### aSyn signature in AD-LB, AD, PDD and control brain tissues

**Fig. 2:**
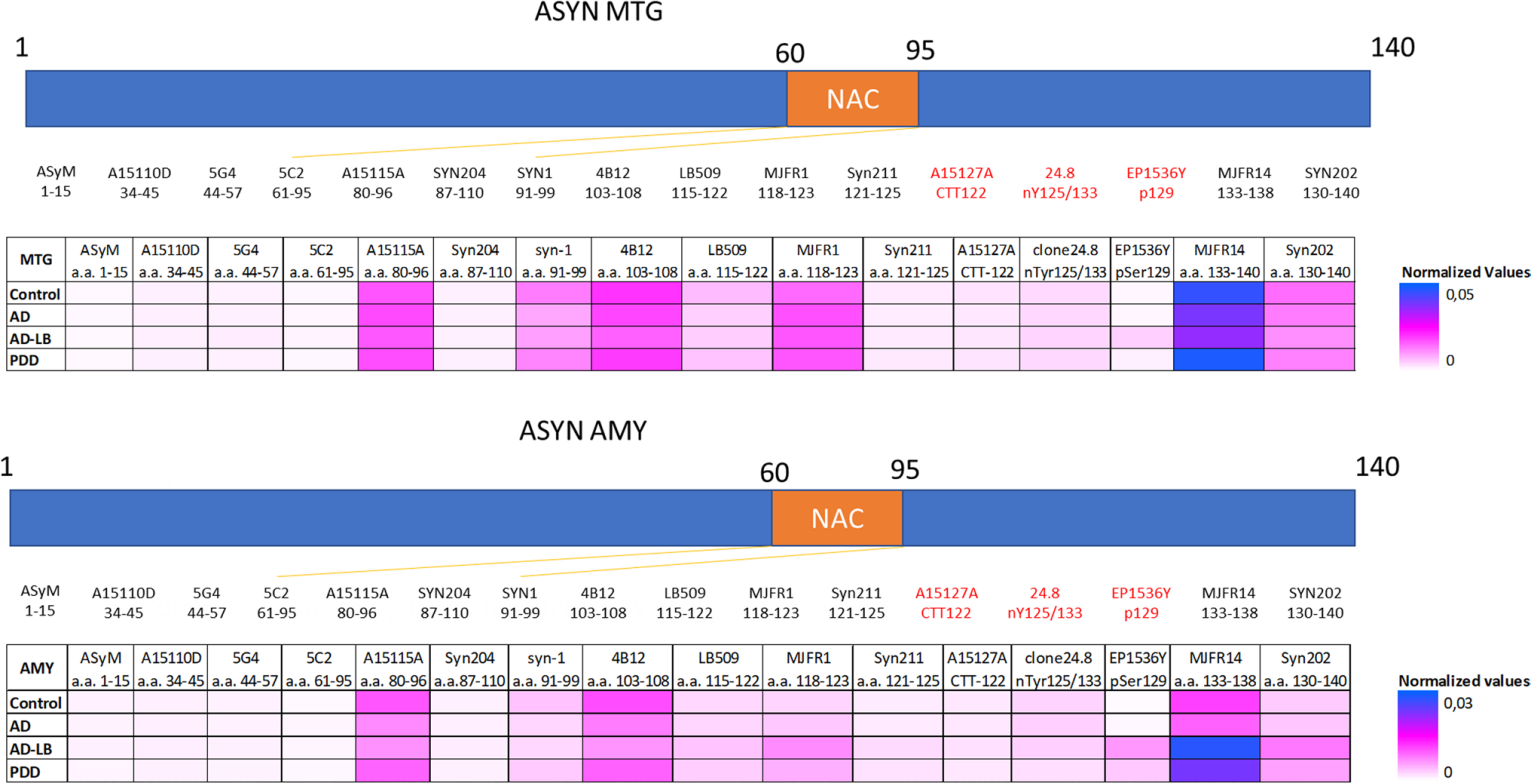
Heatmaps depicting epitope mapping of aSyn in the human brain. MJFR14 (a.a. 133–138) showed best overall binding to both the MTG and AMY brain tissues and showed increased detection of aSyn in the AMY of AD-LB and PDD cases when compared to controls and AD cases. C-terminal antibodies LB509, MJFR1, Syn211 and Syn202 detected also more aSyn in the AD-LB cases when compared to controls and/or AD cases in the AMY. EP1536Y detected more pS129 aSyn in the MTG and AMY of AD-LB cases compared to controls and AD cases, while only detecting more pS129 aSyn in the AMY when comparing PDD to controls cases. Each tile displays the averaged normalized value per group, as is depicted by the scale bar. PTM-specific antibodies are highlighted in red. *NAC* non-amyloid-β component region, *CTT *C-terminal truncation site, *n *nitration site, *p* phosphorylation site, *a.a.* amino acid

We tested 16 aSyn antibodies (Fig. 2) targeting epitopes spanning the N-terminal domain to the C-terminal as well as PTMs (phosphorylation, truncation, and nitration) for MTG and AMY tissue of all cases.

In the MTG (Fig. 2), antibodies targeting the NAC-region and the end of the C-terminus showed best binding to brain homogenates, with MJFR14 (a.a. 133–138) being the best binder. Syn 204 (a.a. 87–110), was an exception, with little binding observed. Antibodies targeting the N-terminal domain, C-terminal truncated and nitrated aSyn showed very minimal to no binding to crude tissue homogenates. No group differences were observed for these antibodies. We observed differences across groups for the Syn-1 and EP1536Y antibody. Syn-1 (a.a. 91–99) showed weaker binding to AD cases compared to controls (*p* = 0.03; Supplementary Fig. S5). EP1536Y (pS129) showed stronger binding in AD-LB cases compared to controls (*p* < 0.01) and AD (*p* < 0.01) cases but no difference was observed between the PDD group and any of the other investigated conditions (*p* > 0.05, Supplementary Fig. S5).

In the AMY (Fig. 2), the binding profile was similar to the MTG. However, in contrast to the MTG, C-terminal antibodies showed significant differences across groups in the AMY. MJFR14 (133–138) showed stronger binding to AD-LB cases when compared to controls (*p* < 0.01) and AD (*p* < 0.01) cases, which was also the case for PDD when compared AD (*p* = 0.02) cases (Supplementary Fig. S6). Despite a weaker binding, similar results were found for the C-terminus binding antibodies LB509 (a.a. 115–122) with stronger binding to AD-LB cases when compared to AD (*p* = 0.03) and control (*p* = 0.02) cases, but no differences between PDD cases and any of the other groups (*p* > 0.05; Supplementary Fig. S6). MJFR1 (a.a. 118–123) also showed stronger binding to AD-LB cases compared to controls (*p* = 0.02; Supplementary Fig. S6). Similarly, Syn211 (a.a. 121–125) showed preferential binding to AD-LB cases over control (*p* < 0.001) and AD (*p* = 0.026) cases as well as in PDD cases over controls (*p* = 0.02). It should be noted that overall binding of Syn211 was very weak (Supplementary Fig. S6). Syn202 (a.a. 130–140) also showed stronger binding to AD-LB cases compared to controls (*p* < 0.01; Supplementary Fig. S6). EP1536Y (pS129) showed stronger binding to AD-LB compared to control (*p* < 0.0001) and AD (*p* < 0.01) groups, while only showing stronger binding in PDD cases when compared to controls (*p* < 0.01). Interestingly, antibodies targeting the NAC-region (apart from Syn204 with no signal) or beginning of the C-terminal domain, showed weaker binding in AD and AD-LB when compared to control and PDD groups. A15115A (a.a. 80–95) showed less binding in AD compared to control (*p* < 0.01) and PDD (*p* = 0.02) groups, while also showing weaker binding in AD-LB when compared to controls (*p* < 0.01) and PDD (*p* = 0.03) cases (Supplementary Fig. S6). Similarly, Syn-1 (a.a. 91–99) showed weaker binding in AD and AD-LB when compared to control (*p* < 0.01 for both comparisons) cases (Supplementary Fig. S6). 4B12 (a.a. 103–108), while targeting the beginning of the C-terminus domain, also showed weaker binding in AD cases when compared to control (*p* < 0.01) cases, and showed less binding in AD-LB cases when compared to control (*p* < 0.001) and PDD (*p* = 0.023) cases (Supplementary Fig. S6).

Overall, we identified a significant upregulation of pS129 aSyn and enrichment of C-terminus epitopes in AD-LB (for the MTG and AMY) and to a lesser extent in PDD (for the AMY only) cases when compared to control and AD cases without aSyn pathology. The very end of the C-terminal proved to be the most available epitope for antibody binding (MJFR14) and was enriched in cases with aSyn pathology. Similarly, pS129 aSyn (EP1536Y) signal was significantly higher in cases with aSyn pathology. Manual pathological scoring of EP1536Y in cryosections showed significant correlations with EP1536Y values as measured with DB (see Supplementary Fig. S2).

### Selected phospho-tau variants are differentially expressed in soluble versus insoluble fractions across groups

**Fig. 3:**
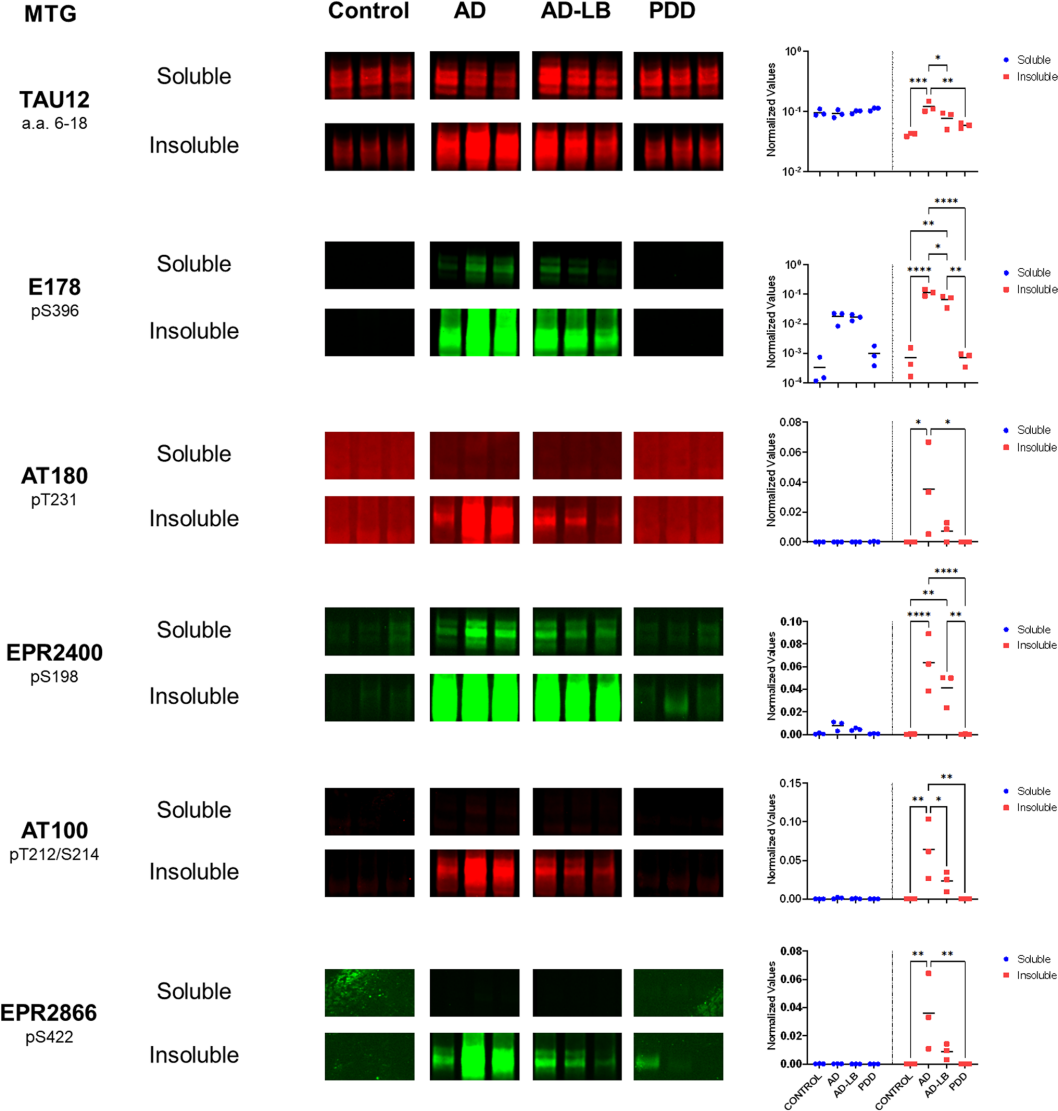
Multiplexed western blots for a subset of tau antibodies revealed distinct differences between soluble and insoluble protein fractions in the MTG. TAU12 detected tau in all investigated groups in both the soluble and insoluble fractions. All investigated pTau species were enriched in the insoluble protein fractions from AD and/or AD-LB cases when compared to controls and PDD cases. pS396 tau (E178) and pS198 tau (EPR2400) were also detected in soluble fractions from AD cases, indicating early involvement of these phosphorylation sites in the multimerization process of tau, while pS396 tau was also enriched in the insoluble fraction of the AD cases without Lewy pathology when compared to AD cases with concomitant pathology

Six antibodies were selected based on their strong binding to brain tissue homogenates and/or group differences in the DB setup for deeper analysis by western blot in the MTG (Fig. 3). TAU12 detected tau in both the soluble and insoluble protein fractions in the MTG of all investigated cases (Fig. 3). There was no significant difference in TAU12 expression between fractions (*p* > 0.05). TAU12 detected more insoluble tau in the AD cases compared to all other groups (vs AD-LB, *p* = 0.04; vs PDD, *p* < 0.01; vs controls, *p* < 0.01). TAU12 detected all six tau isoforms, as was confirmed by detection of the tau ladder, and did not show any other bands, indicating high specificity for tau (Supplementary Fig. S7).

Our investigation of pTau antibodies has revealed interesting differences in the detection of soluble and insoluble tau species. The E178 antibody showed clear detection of pS396 tau in the soluble fractions of AD-LB and AD cases, while showing little detection in soluble fractions of PDD and control subjects. In the insoluble fraction, more pS396 tau was detected in the AD cases compared to all other groups (vs AD-LB, *p* = 0.01; vs PDD, *p* < 0.0001; vs controls, *p* < 0.0001) and more pS396 tau was detected in the insoluble fraction versus the soluble fraction, as highlighted by an interaction between values measured and type of fraction (*p* < 0.0001). Furthermore, more pS396 tau was detected in AD-LB cases compared to PDD cases (*p* < 0.01) and controls (*p* < 0.01). A higher molecular weight species was also detected in the insoluble fraction of all cases (Supplementary Fig. S7). Similar results were found for EPR2400, which was able to detect pS198 tau in both the soluble and insoluble fractions of AD and AD-LB cases but showing barely any signal in controls and PDD cases (Fig. 3). EPR2400 detected significantly more pS198 tau in the insoluble fraction of AD cases when compared to PDD (*p* < 0.0001) and control (*p* < 0.0001) cases. Similarly, more pS198 tau was detected in the insoluble fractions versus the soluble fractions, as highlighted by interaction between values measured and type of fraction (*p* < 0.001).

In contrast, other pTau antibodies showed specific detection of phosphorylated tau species only in the insoluble fractions of AD and AD-LB cases. AT180 detected almost exclusively pT231 tau in the insoluble fraction of AD, showing significantly more detection compared to PDD (*p* = 0.02) and control (*p* = 0.02) cases, and was only limitedly detected in AD-LB cases. Similar findings were detected for AT100, showing almost exclusive detection of pT212/S214 tau in the insoluble fraction of AD cases, showing higher detection compared to all other groups (vs AD-LB, *p* = 0.048; vs PDD, *p* < 0.01; vs controls, *p* < 0.01), while pT212/S214 was also detected in AD-LB cases but to a much lesser extent. An almost identical pattern was seen for EPR2866; pS422 tau was almost exclusively detected in the AD cases, showing higher values in the AD cases compared to PDD (*p* < 0.01) and control (*p* < 0.01) cases, while again pS422 tau was also detected in the AD-LB group but to a much lesser extent. As to be expected from the described results, AT180 (*p* = 0.043), AT100 (*p* < 0.01) and EPR2866 (*p* = 0.016) all showed an interaction between values measured for each antibody and type of fraction measured.

In summary, these results highlight that the N-terminal domain is available for binding in soluble and insoluble tau for all six tau isoforms and that there is no preference of binding to either fractions under denaturing conditions. Interestingly, we detected pS198 and pS396 in the soluble protein fraction of AD cases but only limitedly in AD-LB cases and not in PDD or control cases. Noteworthy, pT212/S214, pT231 and pS422 tau, were selectively detected in the insoluble fractions. All full-length blots of total protein stained, destained and immunostained blots can be found in the supplementary file (Supplementary Fig. S7).

### Phosphorylated S129 alpha-synuclein signal is elevated in insoluble fraction of AD-LB versus PDD cases

**Fig. 4:**
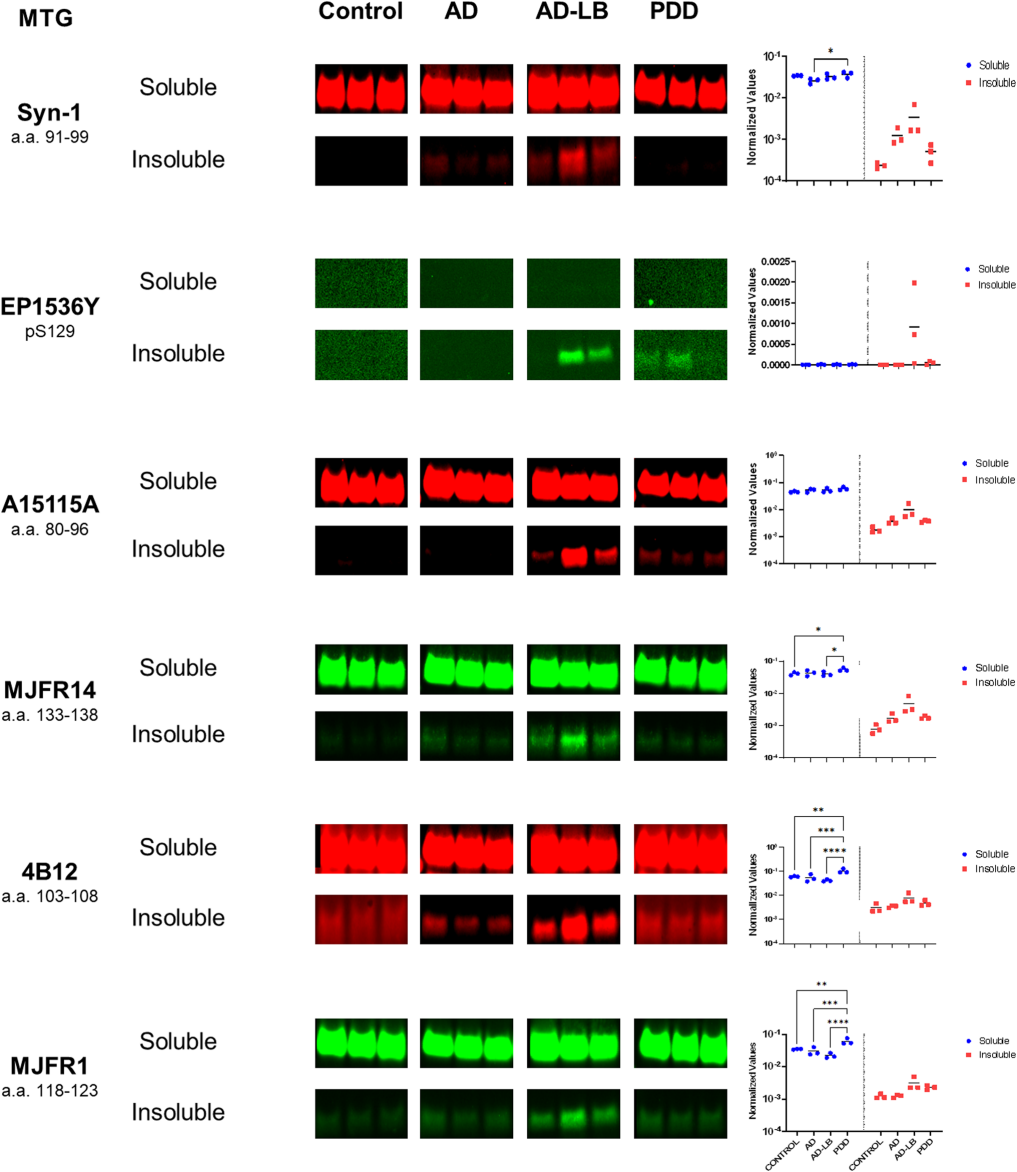
Multiplexed western blots for aSyn in soluble and insoluble fractions of the MTG. All antibodies, except EP1536Y targeting pS129 aSyn, showed strong detection of aSyn in the soluble fractions of all cases. Interestingly, Syn-1 showed stronger detection of soluble aSyn in the soluble fraction of PDD cases compared to all other groups. EP1536 signal was only detected in the insoluble fractions of 2/3 AD-LB cases and very minimally in 2/3 PDD cases, while no signal was detected in any of the other groups. All other antibodies were also able to detected aSyn mainly in the insoluble fraction of the AD-LB cases and to a very limited extent in all other groups

**Fig. 5:**
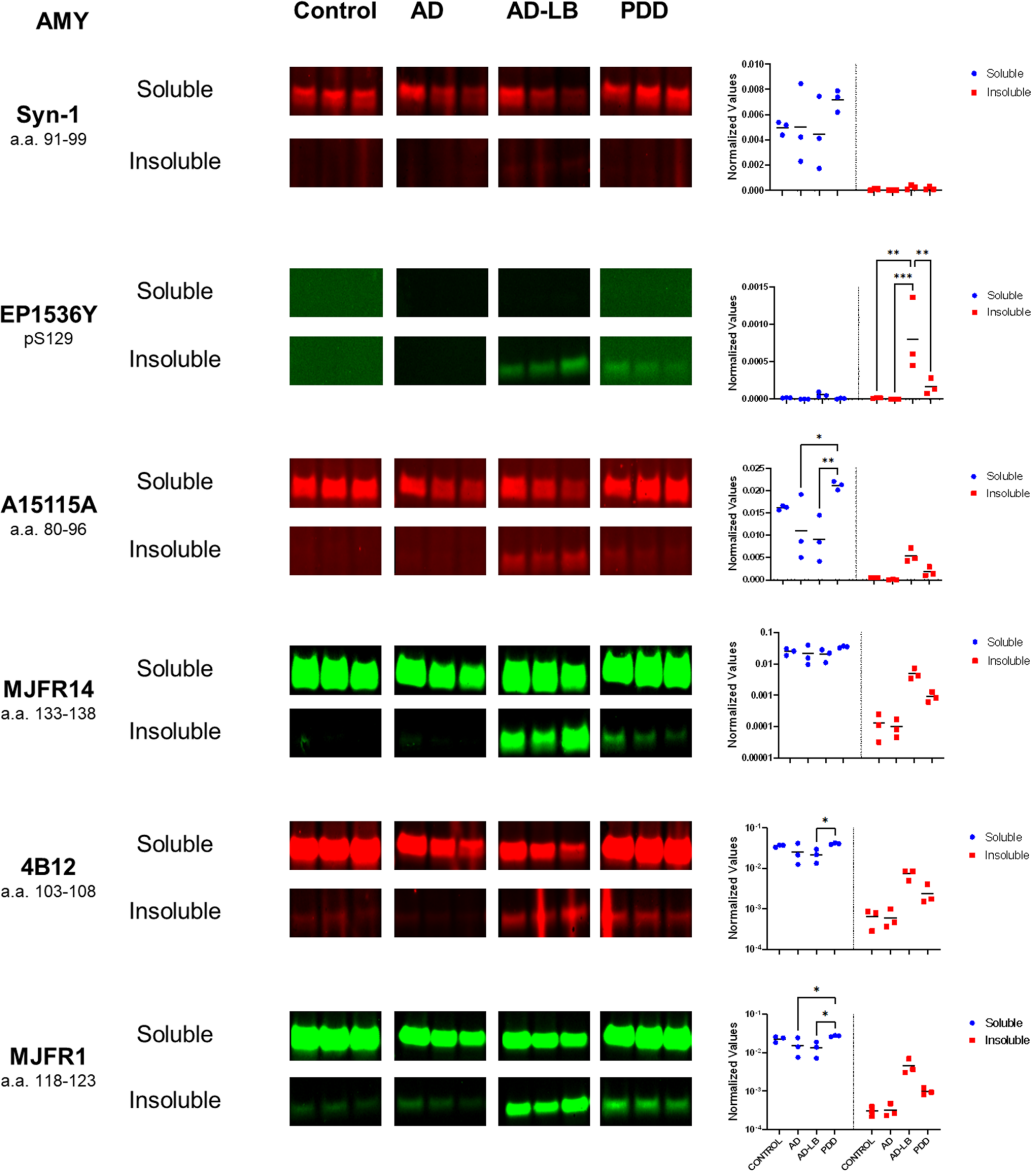
Multiplexed western blots for aSyn in soluble and insoluble fractions of the amygdala. All antibodies, except EP1536Y targeting pS129 aSyn, showed strong detection of aSyn in the soluble fractions of all cases. Syn-1, showed stronger detection of soluble aSyn in the soluble fraction of PDD cases compared to the AD group. Meanwhile, A15115A, 4B12 and MJFR1 detected less soluble aSyn in AD and/or AD-LB cases compared to the other groups. pS129 aSyn (EP1536Y) was significantly upregulated in the insoluble fractions of AD-LB cases compared to all other groups. MJFR14 specifically detected insoluble aSyn in AD-LB and PDD cases, while MJFR1 showed additionally also slight detection of insoluble aSyn in controls and PDD cases

Similarly, six aSyn antibodies which showed strong binding to brain tissue homogenates in DB were chosen for western blot (*n* = 3 per group) in the MTG (Fig. 4) and AMY (Fig. 5). All antibodies except EP1536Y detected more aSyn in the soluble MTG compared to the insoluble fractions (Fig. 4). EP1536Y detected pS129 aSyn predominantly in AD-LB cases and to a much lesser extent in the PDD cases, while detecting no pS129 aSyn in any of the soluble fractions or in the insoluble fractions of the controls and AD cases (Fig. 4). All selected antibodies were also able to detect insoluble aSyn in the AD-LB cases, and to a much lesser extent in the PDD cases (Fig. 4). Syn-1 detected more soluble aSyn in PDD compared to AD (*p* = 0.01) cases. Similarly, MJFR14 detected soluble aSyn in PDD cases compared to AD-LB (*p* = 0.03) and control (*p* = 0.048) cases. Likewise, 4B12 detected more soluble aSyn in PDD cases compared to all other groups (vs AD-LB, *p* < 0.0001; vs AD < 0.001; vs controls, *p* < 0.01).

In the AMY, again all antibodies except EP1536Y detected more aSyn in the soluble fraction compared to the insoluble fractions (Fig. 5). EP1536Y detected more pS129 aSyn in the insoluble fractions of the AD-LB group compared to all other groups (vs PDD, *p* < 0.01; vs AD, *p* < 0.001; vs controls, *p* < 0.01) while also detecting limited pS129 aSyn in the insoluble fraction of the PDD cases (Fig. 5). A15115A (a.a. 80–96) detected significantly more soluble aSyn in PDD cases compared to AD-LB (*p* < 0.01) and AD (*p* = 0.02) cases (Fig. 5). 4B12 (a.a. 103–108) detected less soluble aSyn in AD-LB cases when compared to PDD (*p* = 0.03) cases, while also detecting insoluble aSyn in AD-LB and PDD cases (Fig. 5). MJFR1 (a.a. 118–123) detected also more soluble aSyn in PDD cases compared to AD (*p* = 0.04) and AD-LB (*p* = 0.01) cases. In addition, MJFR1 showed clear detection of insoluble aSyn in AD-LB cases and PDD cases, even very minimally in controls and AD cases (Fig. 5). MJFR14 (a.a. 133–138) showed strong detection of soluble aSyn in all cases and detected insoluble aSyn in AD-LB cases and to a lesser extent in PDD cases (Fig. 5). However, while all antibodies showed no other significant bands, MJFR14 clearly detected a band at approximately 50 kD, indicative of non-specific binding to another protein, as it was both seen in the soluble and insoluble protein fractions of nearly all cases (Supplementary Figs. S8, S9).

These results highlight abundant aSyn pathology in the AMY in AD-LB cases as indicated by the high levels of detected insoluble aSyn. Counterintuitively, we detected more soluble aSyn in PDD cases compared to other groups, as is highlighted by multiple antibodies with varying epitopes over the NAC-region (Syn-1 and A15115A) and C-terminus (4B12 and MJFR1). All full-length blots of total protein stained, destained and immunostained blots can be found in the supplementary file (Figs. S8, S9).

### Similar phospho-tau load in MTG and AMY of AD-LB and AD cases

**Fig. 6:**
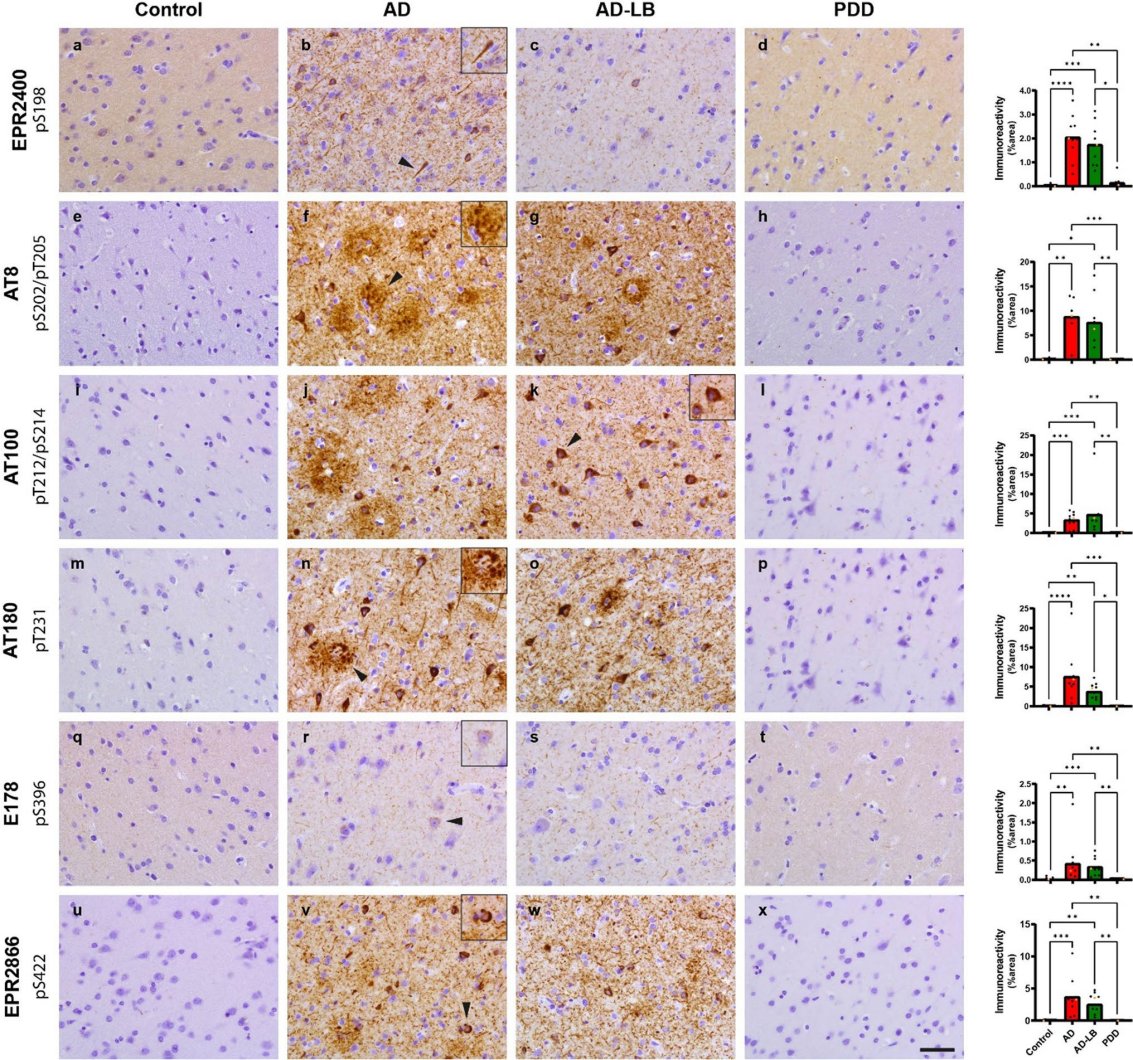
Representative images of immunostained MTG sections with PTM-specific tau antibodies. EPR2400 revealed synaptic staining in controls and PDD sections (**a**, **d**) and many neurofibrillary tangles (NFTs) in AD and AD-LB sections (**b**, **c**). While AT8 detected showed no immunoreactivity (IR) in controls and PDD cases (**e**, **h**), many neuritic plaques, neuropil threads and NFTs were detected in AD and AD-LB cases (**f**, **g**). AT100 also showed no IR in controls and PDD cases (**i**, **l**) while detecting numerous neuritic plaques and neuropil threads in AD cases (**j**) and many NFTs and neuropil threads in AD-LB cases (**k**). No IR was again detected by AT180 in controls and PDD cases (**m**, **p**), while many neuritic plaques, NFTs and neuropil threads were detected in AD and AD-LB cases (**n**, **o**). E178 detected little synaptic staining in all cases (**q**–**t**), while detecting numerous neuropil threads and few NFTs in AD and AD-LB cases (**r**, **s**). EPR2866 showed no IR in PDD and controls subjects (**x**, **u**) while detecting numerous neuropil threads, NFTs and neuritic plaques in AD and AD-LB cases (**v**, **w**). Arrows indicate typical aSyn structures that are displayed in the inserts. The images have been captured at ×400 magnification and the scale bar represents 50 μm

Immunostaining for pTau variants in the MTG detected a significant higher tau load in AD-LB and AD compared to PDD and control cases (Fig. 6). The tau antibody AT8 (pS202/pT205) showed highest immunoreactivity in AD-LB and AD of all investigated antibodies, detecting many NFTs, neuropil threads and neuritic plaques (Fig. 6). Similar pathologies were detected by AT100 (pT212/pS214), AT180 (pT231) and EPR2866 (pS422) in AD-LB and AD cases (Fig. 6). No neuritic plaques were detected with EPR2400 (pS198) and E178 (pS396). While EPR2400 showed both detection of NFTs and neuropil threads, E178 on the other hand showed preferential detection of neuropil threads and only detected few NFTs (Fig. 6).

No significant differences in tau load were seen between AD-LB and AD cases for any of the investigated antibodies. While almost all antibodies showed a slight non-significantly higher tau load in AD versus AD-LB cases, AT100 detected a slightly non-significant higher tau load in AD-LB compared to AD cases (Fig. 6). Interestingly, while AT8, AT100, AT180 and EPR2866 detected neuritic plaques, both EPR2400 and E178 did not show any plaques. Performance of scripts for determining tau immunoreactivity can be found in Supplementary Fig. S10.

### Higher alpha-synuclein load in AD-LB cases versus PDD cases

**Fig. 7:**
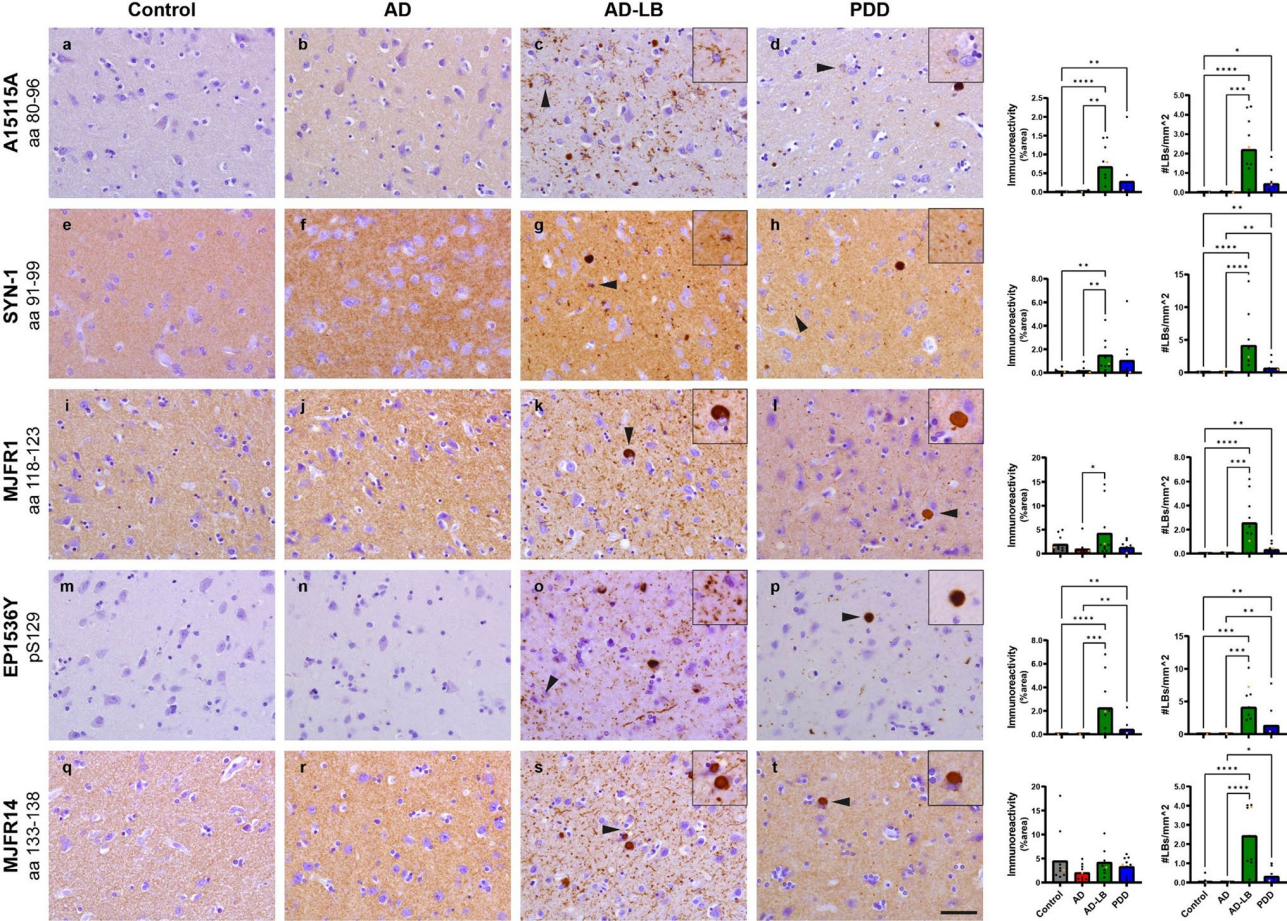
Representative images of immunostained MTG sections with epitope- or PTM-specific aSyn antibodies. A15115A showed no immunoreactivity (IR) in controls cases (**a**), while revealing slight detection of synaptic staining in AD, AD-LB and PDD cases (**b**–**d**) and revealing numerous astrocytic star-shaped inclusions, Lewy neurites (LNs), cortical Lewy bodies (LBs) and diffuse cytoplasmic neuronal immunoreactivity (DCNIR) in AD-LB cases (**c**) and to a lesser extent in PDD cases (**d**). Syn-1 revealed synaptic staining in all cases (**e**–**h**) and revealed similar morphologies in AD-LB and PDD cases as A15115A but to a lesser extent (**g**, **h**). MJFR1 also detected synaptic staining in all cases (**i**–**l**), while detecting numerous LNs and some LBs in AD-LB cases (**k**) and limitedly in PDD cases (**l**). EP1536Y showed no IR in controls and AD cases (**m**, **n**) but detected numerous LNs and LBs in AD-LB cases, while also detecting neuritic plaques (**o**). Only a few LBs and LNs were detected by EP1536Y in PDD cases (**p**). MJFR14 detected synaptic staining in all cases (**q**–**t**) and detected abundant LBs and LNs in AD-LB cases (**s**), while showing limited LBs and LNs in PDD cases (**t**). Arrows indicate typical aSyn structures that are displayed in the inserts. The images have been captured at ×400 magnification and the scale bar represents 50 μm

**Fig. 8:**
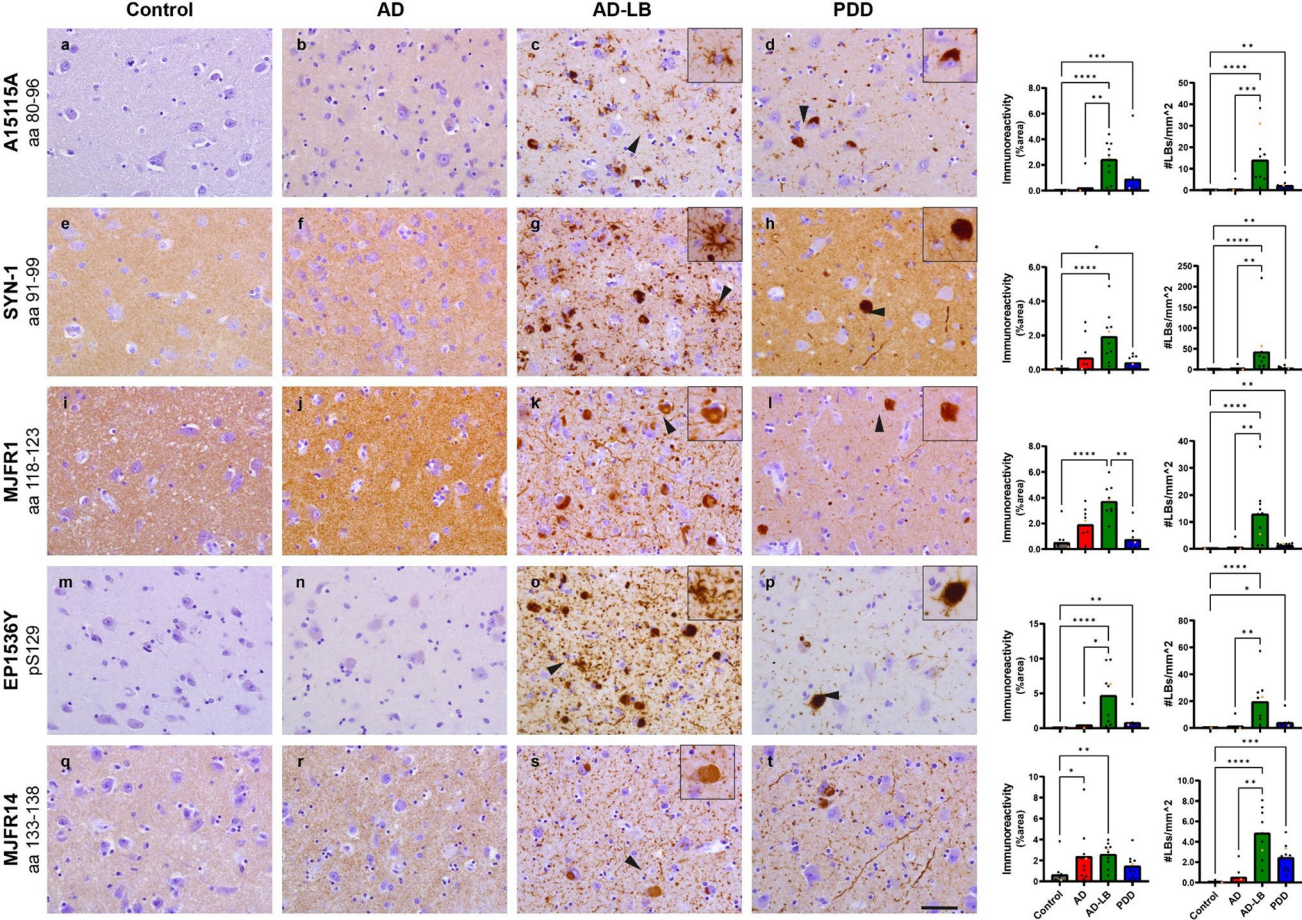
Representative images of immunostained AMY sections with epitope- or PTM-specific aSyn antibodies. A15115A revealed no immunoreactivity (IR) in controls cases (**a**), while revealing slight synaptic staining in AD, AD-LB and PDD cases (**b**–**d**). A15115A revealed typical astrocytic star-shaped inclusions in AD-LB cases (**c**) and somatic inclusions in PDD cases (**d**). SYN-1 revealed synaptic staining in all groups (**e**–**h**), while also revealing astrocytic aSyn inclusions in AD-LB cases (**g**) and LNs as well as cortical LBs in PDD cases (**h**). MJFR1 detected synaptic staining in all groups (**i**–**l**) and additionally diffuse aSyn inclusions in AD-LB and PDD sections (**k**, **l**) while also detecting abundant LBs and LNs. EP1536Y showed no IR in both controls and AD cases (**m**, **n**) while revealing numerous aSyn inclusions in AD-LB cases, including neuritic plaques (**o**) in AD-LB cases and granular LBs (**p**) in PDD cases. MJFR14 revealed synaptic staining all groups (**q**–**t**) and DCNIR in AD-LB cases next to numerous LNs and LBs in both AD-LB and PDD cases (**r**, **t**). Arrows indicate typical aSyn structures that are displayed in the inserts. The images have been captured at ×400 magnification and the scale bar represents 50 μm

Overall, a higher aSyn load was observed in AD-LB cases compared AD and control cases for all investigated (*n* = *6*) antibodies (Figs. 7, 8). The same applied for the PDD cases, which also consistently showed higher aSyn loads compared to AD and control cases (Figs. 7, 8). In addition, MJFR1 (118–123) showed higher immunoreactivity in AD-LB cases compared to PDD (*p* < 0.01) within the AMY (Fig. 8).

We observed a wide range of detected morphologies, which were antibody and sometimes group dependent. A15115A (a.a 80–96) showed no immunoreactivity in controls and some synaptic staining in AD cases. LBs, diffuse cytoplasmic neuronal immunoreactivity (DCNIR), LNs and star-like/astrocytic inclusions were detected with A15115A in AD-LB cases and to a lesser extent in PDD cases (Fig. 7). Similar morphologies were detected by Syn-1 (a.a. 91–99) in AD-LB and PDD cases, (Fig. 7). MJFR1 detected many LBs and LNs in AD-LB and to a lesser extent in PDD cases (Fig. 7). Meanwhile, EP1536Y (pS129) immunoreactivity was exclusively detected in AD-LB and PDD cases, showing numerous LBs and LNs in AD-LB and only very limitedly in PDD cases (Fig. 7). EP1536Y also revealed neuritic plaques in AD-LB cases (Fig. 7). MJFR14 (a.a. 133–138) revealed numerous LBs and LNs in AD-LB and to a much lesser extent in PDD cases (Fig. 7). All antibodies, except EP1536Y, showed synaptic (background) staining in nearly groups (Fig. 7).

Similar morphologies were detected in the AMY (Fig. 8). However, aSyn pathology was much more abundant in AMY compared to the MTG of AD-LB and PDD cases. Antibodies targeting the NAC-region (A15115A and Syn-1) detected numerous astrocytic star-shaped inclusions in predominantly AD-LB and only limitedly in PDD cases (Fig. 8). These type of inclusions were not observed for any of the investigated C-terminal antibodies, including EP1536Y (Figs. 7, 8). Synaptic (background) staining was higher in AD versus control cases for all antibodies except EP1536Y (Figs. 7, 8). DCNIR was revealed in AD-LB and PDD cases by all investigated antibodies (Figs. 7, 8). For both the MTG and AMY, NAC-region antibodies (Syn-1 and A15115A) predominantly detected astrocytic star-shaped pathology in AD-LB cases and only minimally in PDD cases (Figs. 7, 8). Performance of scripts for determining aSyn immunoreactivity and LB detection can be found in Supplementary Figs. S11 and S12.

### Phospho-tau and alpha-synuclein co-localize within the same astrocytes of AD-LB cases in the MTG

**Fig. 9:**
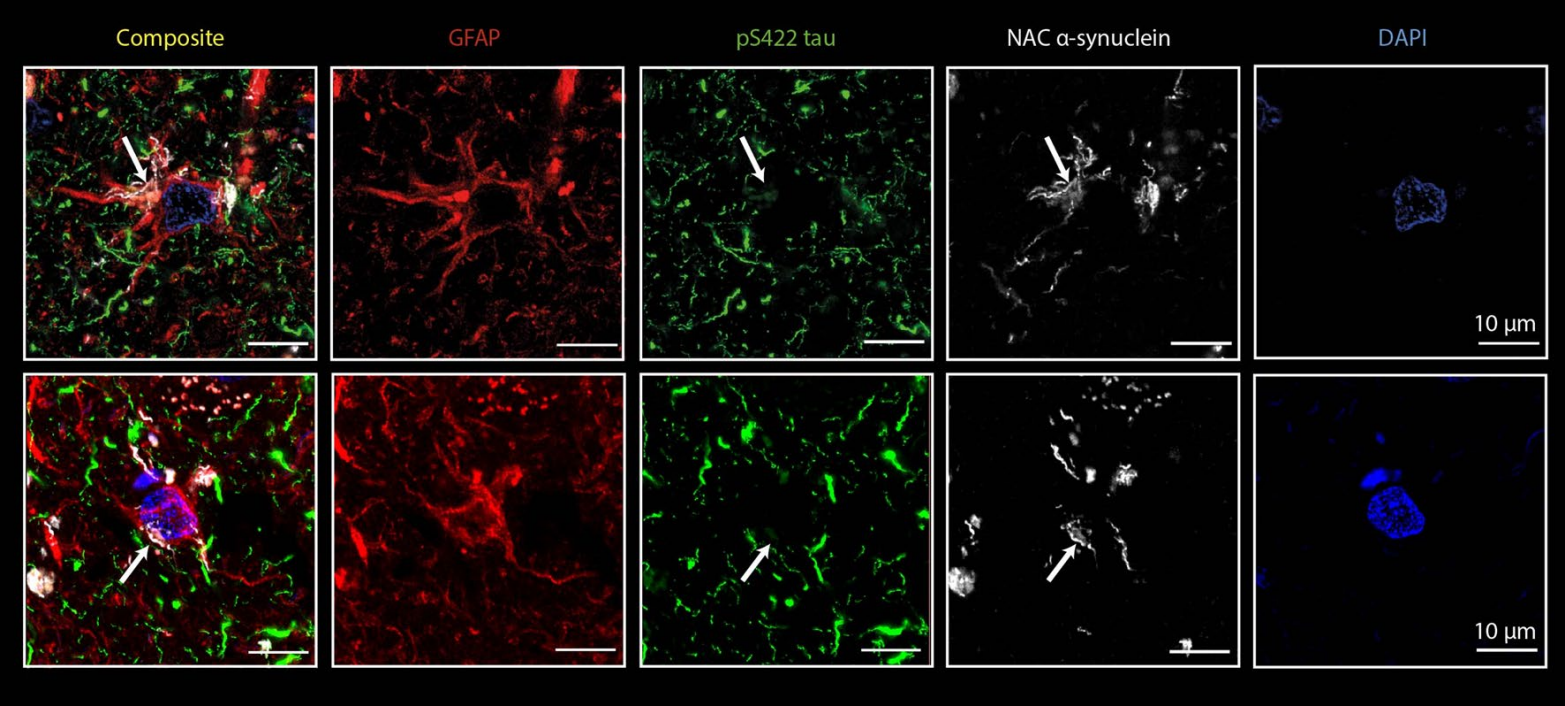
pTau and aSyn co-localize within the same astrocytes in the MTG of AD-LB cases. Multi-labeling revealed co-localization (arrows) of pS422 tau (green) and NAC-region aSyn (white) within the same astrocytes (red), which could suggest possible tau/aSyn co-aggregation. Co-localization was primarily observed within the cell body and to a lesser extent in some of the processes of the astrocytes. Each row shows co-localization in a different AD-LB case. DAPI was used as counterstain to visualize the nuclei

To assess whether the star-shaped aSyn positive astrocytes contained any concomitant tau pathology, a triple staining was performed for GFAP, pS422 tau (EPR2866) and aSyn (A15115A). By examining the MTG in multiple AD-LB cases, we detected co-localization of pS422 tau and NAC-region aSyn within the same astrocyte (see Fig. 9). This could be seen in multiple astrocytes within the same case (results not shown). We confirmed these findings by examining multiple cases and observed the same phenomenon in another AD-LB case within the MTG (see Fig. 9). Co-localization seemed to primarily occur within the cell body and to a lesser extent within the processes (see Fig. 9).

## Discussion

**Fig. 10:**
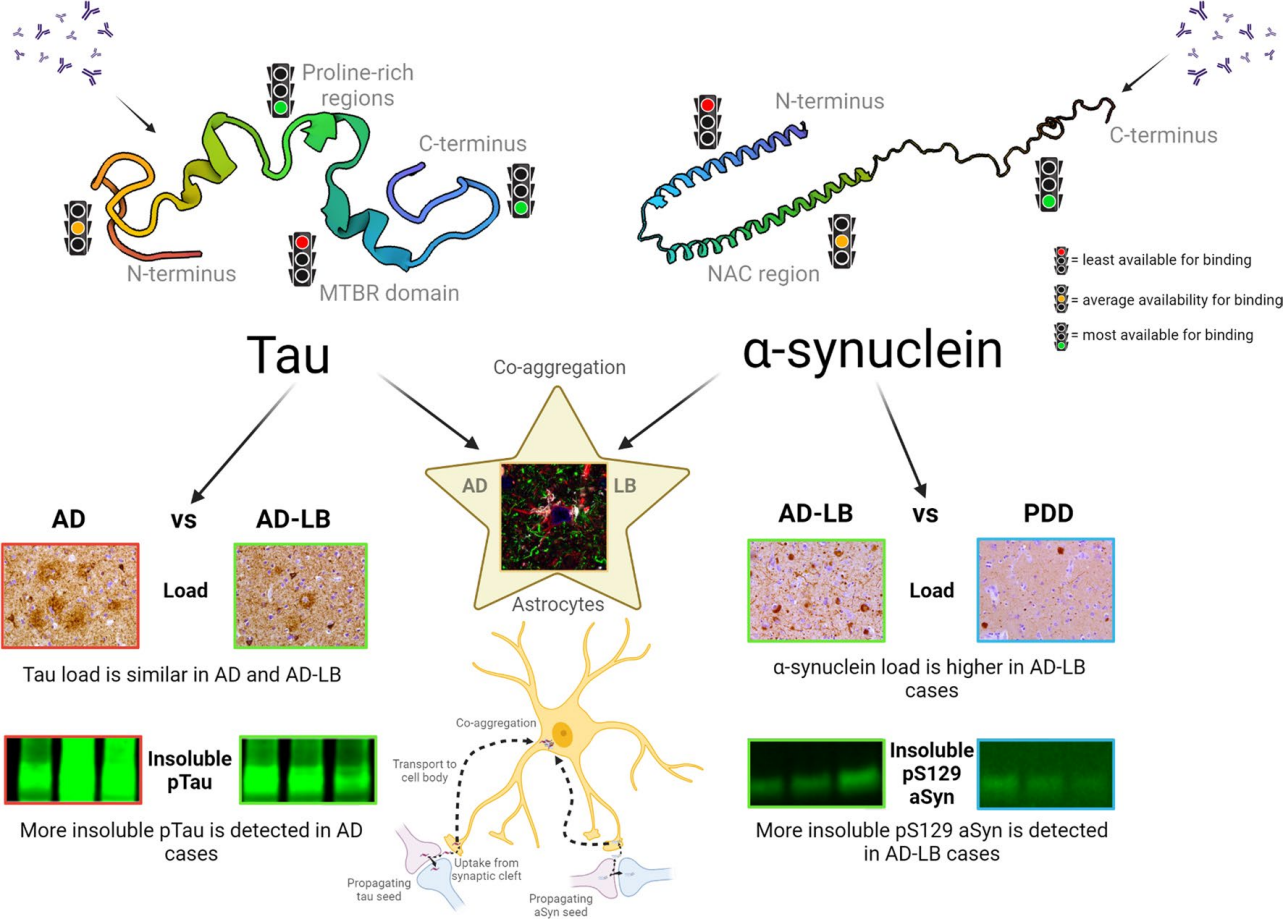
Schematic overview highlighting available epitopes on tau and alpha-synuclein for antibody binding in the human brain under non-denaturing conditions, distinct tau and aSyn profiles in pure versus mixed cases and tau/aSyn co-aggregates in astrocytes in AD-LB cases. This study highlights the proline-rich regions and C-terminus of tau and the C-terminus of aSyn to be the most available for antibody binding in detergent-free crude human brain homogenates. These regions were also characteristic for pathological variants of both proteins, as preferential binding was seen in AD/AD-LB cases for tau and AD-LB/PDD cases for aSyn. Differences were observed in the molecular profile between AD-LB and AD cases for tau, as tau load was similar (AT8) while more insoluble pTau (pS396) was detected in AD versus AD-LB cases. Differences were also observed in the molecular aSyn profile between AD-LB and PDD cases, as both aSyn load (MJFR1) was higher and an increased detection of insoluble pS129 aSyn was seen in AD-LB versus PDD cases. Co-localization between pTau (pS422) and aSyn (NAC-region) could be detected in astrocytes (GFAP) in the MTG of AD-LB cases. A theoretical model highlights the uptake of tau and aSyn seeds from the tripartite synapse, which leads to translocation of these seeds to the cell body for targeted degradation, after which impartial degradation results in the formation of co-aggregates. Created with BioRender.com

In the current study, we identified domains of the tau and aSyn protein which are both available for antibody binding under non-denaturing conditions in the human brain and reveal pathological proteoforms in AD-LB, AD and PDD cases. For tau, parts of the proline-rich regions and C-terminus are available for antibody binding and characteristic for AD-LB and AD cases. For aSyn, the end of the C-terminus was most available for antibody binding and characteristic for AD-LB and PDD conditions. Both pTau and aSyn species detected pathological proteoforms, as pTau was significantly upregulated in AD-LB and AD cases and phospho-aSyn in AD-LB and PDD cases. We also showed differential expression of pTau species in soluble and insoluble fractions; pS198 and pS396 tau was specifically detected in the soluble fraction of AD-LB and AD cases but not in PDD and control cases, suggestive of early involvement of these PTMs in the aggregation process of tau. We also showed a higher pathological aSyn load in AD-LB versus PDD cases, while pathological tau load seems to be similar or even slightly lower in AD-LB versus AD cases. Finally, aSyn antibodies targeting the NAC domain specifically detected astrocytic star-shaped pathology which were predominantly detected in the AD-LB cases. These results provide an increased understanding of the underlying pathological heterogeneity in the neurodegeneration spectrum (Fig. 10).

For tau, the N-terminal antibody TAU12 (a.a. 6–18) showed best binding to brain homogenates overall. However, TAU12 binding was lower in AMY homogenates for the AD and AD-LB cases compared to PDD and control cases, suggestive of this epitope being characteristic for physiological forms of tau. This might explain the lack of clinical effects of therapeutic antibodies targeting the N-terminal domain, as this domain might be less available for antibody binding in pathological propagating tau species [27]. Moreover, tau has been described to be heavily truncated in the N-terminus in AD, which could explain the lower signal for TAU12 in the AMY in AD cases, as these pathological tau fragments might not be detected by a N-terminal antibody [30]. Meanwhile, we showed that proline-rich regions (HT7:159–163 and Tau5:210–230) and the C-terminus (TAU46:401–441) tau antibodies bind preferentially to MTG and AMY tissue homogenates of AD and AD-LB donors over controls and PDD cases. In line with these findings, a recent study has found a higher ratio of high molecular weight tau species to low molecular weight tau species for antibodies targeting the C-terminal domain versus the N-terminal domain, indicating that antibodies targeting this domain are more likely to bind to aggregated tau species [75]. In addition, we measured very little truncated tau at residue at 421, questioning the importance of this specific modification in relation to AD pathology [10, 24, 39]. In concordance, a recent study found only an increase in truncation 421 in low molecular weight tau in the AD brain but not in high molecular weight tau, suggesting that this modification is most likely not an important driver of tau aggregation in AD [75].

For aSyn, non-phospho epitopes on the C-terminus were most available for antibody binding and bound preferentially to AMY homogenates from AD-LB and PDD cases over AD and control cases, whereas the N-terminus was barely available for antibody binding under non-denaturing conditions. Since aSyn is predominantly a membrane binding protein involved in docking of synaptic vesicles and the N-terminal region being the membrane binding region, it could explain the lack of binding of antibodies targeting this region considering the interactions with lipids and possible lack of any binding pocket in the alpha-helical configuration of this domain [9]. This might explain the recent failure of N-terminal therapeutic antibody cinpanemab (1–10) to meet the primary or secondary endpoints, after which development was halted [37]. An alternative explanation could be that the affinity of these antibodies was simply too weak to detect aSyn in our human brain samples, as a low affinity for antibody 5G4 (a.a. 44–57) has been described for binding to recombinant aSyn monomers and aggregates [36]. Many of the therapeutic antibodies which are currently being tested in clinical trials are predominantly targeting the C-terminus and considering our findings, this strategy might be a more fruitful approach [20]. However, it should be noted that truncation could play an important role in the aggregation process of aSyn and antibodies targeting the end of the C-terminus might not bind to pathological protein species which are truncated in the C-terminus [61]. Conversely, truncation at residue 122 was barely detected in the current study, highlighting the need for more research confirming or refuting the importance of aSyn truncation in the pathogenesis and progression of synucleinopathies.

Phosphorylated tau and aSyn species were characteristic for insoluble protein variants, which is in line with the observation that tau aggregates are hyperphosphorylated and the predominant modification of aSyn in LBs being phosphorylation at Serine 129 [15, 51]. More interestingly, we identified specific phosphorylated species of tau (pS198 and pS396) in the soluble protein fractions of both AD-LB and AD cases. This notes the involvement of these modifications in the early multimerization process of tau, as beta-sheet rich aggregated tau oligomers require harsher buffer solutions in order to be fully dissolved and are thus likely not present in the soluble protein fraction. Previously, pTau species pS198, pS199 and pS416 have been shown to correlate with early tau multimerization in the hippocampus, temporal cortex and entorhinal cortex in AD [19]. While pS396 seems to be elevated in only late Braak stages (V–VI), levels do seem to correlate with oligomerization state of tau in the hippocampus [19, 49]. In a study comparing multiple tau proteoforms across oligomeric (O-tau), sarkosyl-insoluble 1 and 2 (SI_1_ and SI_2_) and heat stable monomeric (HS-tau) tau fractions from AD brains, pS396/404 (PHF-1 antibody) expression was similar across O-tau, SI_1_ and SI_2_ fractions but not detected in HS-tau, again suggestive of pS396 being involved early in tau multimerization [38]. Intriguingly, we did not find an upregulation of pT231 in the soluble fraction of AD cases, although this phospho species has been described as being an early modification in the aggregation process of tau [5, 19, 49]. A stoichiometric tau fibrillation model based on quantitative proteomic data, highlighted the 0N and 4R tau isoforms to be predisposed for aggregation and suggested phosphorylation of 6 PRR residues and S396 to be one of the initiators for tau aggregation, since these were specifically increased in a cluster of subjects with an average Braak NFT stage III–IV [70]. Overall, our results and others suggest that specific phosphorylation sites in the PRR and C-terminus seem to be critical for tau aggregation.

Here, we highlight a higher aSyn load in AD-LB compared PDD cases as seen within the AMY using the MJFR1 (a.a. 118–123) antibody. An earlier study investigating tau, Aβ and aSyn co-pathologies in a neuropathologically defined cohort of mixed AD/Dementia with Lewy bodies (DLB) and pure AD and DLB cases did not report a significant difference in aSyn load between mixed versus pure groups [68]. However, it should be noted that this study used a different antibody to detect aSyn (Chemicon, AB5038) and, although not significant, the study did note a higher pathological aSyn load in the AMY of mixed AD-LB compared to pure DLB cases [68]. Another study showed that aSyn burden was also higher in all neocortical regions of PD cases with AD co-pathology compared to cases without AD pathology, while similar to our findings, tau load was higher in AD cases compared to mixed AD cases, except in the superior temporal cortex [14]. These results along with our own, suggest that aSyn aggregation is aggravated in the presence of tau pathology in the AMY. Earlier studies highlight the secondary formation of LBs in the presence of tau pathology in the AMY in brain tissue from various tauopathies [53]. This phenomenon could be possible explained by liquid–liquid phase separation (LLPS) of aSyn forming into liquid droplets consisting of biological polymers (proteins and RNA), eventually transforming into solid-like hydrogels rich in oligomers and fibrillary species [55]. While tau undergoes LLPS by itself under physiological conditions, recent in vitro studies show that aSyn does not, but does so in the presence of tau [48, 60, 69]. In addition, while Cdk2-phosphorylation of tau increases tau concentration in RNA-induced droplets, it reduces the amount of aSyn in the droplets [60]. This could possibly explain the higher pTau levels in pure AD cases compared to AD-LB; since aSyn most likely forms coacervate droplets in the presence of tau, hyperphosphorylation of tau on the P2 region would interfere with aSyn-tau interactions [60]. It should be noted that the AMY is especially prone for developing aSyn pathology in Lewy body dementia cases [63]. Brainstem regions, which have not been evaluated in the current study, might show differences in terms of aSyn burden for AD-LB and PDD cases.

To the best of our knowledge, this is the first study which shows co-localization of pTau and aSyn within the same astrocytes. Accumulating evidence suggests a stronger involvement of astrocytes in the pathogenesis and progression both AD and PD than previously thought of [21, 25, 33, 62]. Astrocytic internalization of propagating seeds from the tripartite synapse has been described for both tau and aSyn and studies suggest impaired astrocytic functioning of the autophagy-lysosomal and ubiquitin–proteasome pathways to result in failed clearance of protein aggregates, triggering microglial activation [21, 25, 33, 62]. Moreover, bidirectional spread between astrocytes and neurons has been reported and astrocytic propagation of seeds via tunneling nanotubes may further accelerate the disease progression [41, 56]. We hypothesize, therefore, that astrocytic end-feet internalize tau and aSyn aggregates from the tripartite synapse, which are being translocated to the soma for subsequent degradation, after which impartial degradation leads to tau/aSyn co-aggregation (Fig. 10). The implications of tau/aSyn co-aggregates in astrocytes for the disease progression and possible mechanisms that drive this co-aggregation should be investigated by future studies.

Strengths of this study include the use a very well defined cohort and the use of multiple tissue types, regions and techniques to assess epitopes/PTMs/isoforms in crude, soluble and insoluble tissue fractions, both under denaturing and non-denaturing conditions. In addition, multiple antibodies were used for immunohistochemistry to capture the full range of tau and aSyn pathologies. However, it also should be noted that our study comes with a few shortcomings. First, while our cohort was well characterized, sample sizes were limited for each group and variability within the groups was relatively high. Furthermore, techniques used here were semi-quantitative and only three cases per group were examined for the western blot experiments. We also noted an AD case (case #14; see Supplementary Table 1) which did display aSyn pathology in the AMY, indicating this case should have been classified as an AD-LB case instead of an AD case. Future research should focus on validating these findings in larger cohorts, adding other PTM-specific antibodies and using more quantitative methods.

In conclusion, we highlight (1) regions on tau and aSyn in the human brain that are available for antibody binding under non-denaturing conditions (2) distinct tau and aSyn molecular profiles for pure and mixed cases and (3) tau/aSyn co-aggregates in astrocytes of AD-LB cases within the MTG (Fig. 10). These data can be used for therapeutic antibody development and support biomarker discovery. Considering converging pathologies in neurodegenerative diseases via molecular crosstalk, therapeutic strategies should have a focused approach on combining different treatment strategies which are targeted against the full spectrum of pathological proteins [58]. This paper outlines the urgent need for getting a better understanding of the neurodegeneration continuum, as clinical and pathological heterogeneity within AD and PD requires us to further investigate what links and separates these disease from one another.

### Supplementary Information

Below is the link to the electronic supplementary material.

Supplementary file1 (DOCX 7808 KB)

## Data Availability

Raw and processed data which support the findings in this article can be shared by the corresponding author upon reasonable request. Data are stored in a controlled access workspace at the Amsterdam UMC.

## References

[1] Alafuzoff I, Arzberger T, Al-Sarraj S, Bodi I, Bogdanovic N, Braak H, Bugiani O, Del-Tredici K, Ferrer I, Gelpi E (2008). Staging of neurofibrillary pathology in Alzheimer’s disease: a study of the BrainNet Europe Consortium. Brain Pathol.

[2] Alafuzoff I, Ince PG, Arzberger T, Al-Sarraj S, Bell J, Bodi I, Bogdanovic N, Bugiani O, Ferrer I, Gelpi E (2009). Staging/typing of Lewy body related alpha-synuclein pathology: a study of the BrainNet Europe Consortium. Acta Neuropathol.

[3] Arawaka S, Sato H, Sasaki A, Koyama S, Kato T (2017). Mechanisms underlying extensive Ser129-phosphorylation in alpha-synuclein aggregates. Acta Neuropathol Commun.

[4] Arnsten AFT, Datta D, Del Tredici K, Braak H (2021). Hypothesis: tau pathology is an initiating factor in sporadic Alzheimer’s disease. Alzheimers Dement.

[5] Augustinack JC, Schneider A, Mandelkow EM, Hyman BT (2002). Specific tau phosphorylation sites correlate with severity of neuronal cytopathology in Alzheimer’s disease. Acta Neuropathol.

[6] Awa S, Suzuki G, Masuda-Suzukake M, Nonaka T, Saito M, Hasegawa M (2022). Phosphorylation of endogenous alpha-synuclein induced by extracellular seeds initiates at the pre-synaptic region and spreads to the cell body. Sci Rep.

[7] Badiola N, de Oliveira RM, Herrera F, Guardia-Laguarta C, Goncalves SA, Pera M, Suarez-Calvet M, Clarimon J, Outeiro TF, Lleo A (2011). Tau enhances alpha-synuclein aggregation and toxicity in cellular models of synucleinopathy. PLoS ONE.

[8] Bankhead P, Loughrey MB, Fernandez JA, Dombrowski Y, McArt DG, Dunne PD, McQuaid S, Gray RT, Murray LJ, Coleman HG (2017). QuPath: open source software for digital pathology image analysis. Sci Rep.

[9] Bartels T, Ahlstrom LS, Leftin A, Kamp F, Haass C, Brown MF, Beyer K (2010). The N-terminus of the intrinsically disordered protein alpha-synuclein triggers membrane binding and helix folding. Biophys J.

[10] Binder LI, Guillozet-Bongaarts AL, Garcia-Sierra F, Berry RW (2005). Tau, tangles, and Alzheimer’s disease. Biochim Biophys Acta.

[11] Brás IC, Khani MH, Vasili E, Möbius W, Riedel D, Parfentev I, Gerhardt E, Fahlbusch C, Urlaub H, Zweckstetter M (2021) Common molecular mechanisms underlie the transfer of alpha-synuclein, Tau and huntingtin and modulate spontaneous activity in neuronal cells. bioRxiv

[12] Chlebowski AC, Kisby GE (2020). Protocol for high-throughput screening of neural cell or brain tissue protein using a dot-blot technique with near-infrared imaging. STAR Protoc.

[13] Colin M, Dujardin S, Schraen-Maschke S, Meno-Tetang G, Duyckaerts C, Courade JP, Buee L (2020). From the prion-like propagation hypothesis to therapeutic strategies of anti-tau immunotherapy. Acta Neuropathol.

[14] Coughlin D, Xie SX, Liang M, Williams A, Peterson C, Weintraub D, McMillan CT, Wolk DA, Akhtar RS, Hurtig HI (2019). Cognitive and pathological influences of tau pathology in Lewy body disorders. Ann Neurol.

[15] Cowan CM, Mudher A (2013). Are tau aggregates toxic or protective in tauopathies?. Front Neurol.

[16] Cummings J, Lee G, Nahed P, Kambar M, Zhong K, Fonseca J, Taghva K (2022). Alzheimer’s disease drug development pipeline: 2022. Alzheimers Dement (N Y).

[17] Dasari AKR, Kayed R, Wi S, Lim KH (2019). Tau interacts with the C-terminal region of alpha-synuclein, promoting formation of toxic aggregates with distinct molecular conformations. Biochemistry.

[18] de Boni L, Watson AH, Zaccagnini L, Wallis A, Zhelcheska K, Kim N, Sanderson J, Jiang H, Martin E, Cantlon A (2022). Brain region-specific susceptibility of Lewy body pathology in synucleinopathies is governed by alpha-synuclein conformations. Acta Neuropathol.

[19] Ercan-Herbst E, Ehrig J, Schondorf DC, Behrendt A, Klaus B, Gomez Ramos B, Prat Oriol N, Weber C, Ehrnhoefer DE (2019). A post-translational modification signature defines changes in soluble tau correlating with oligomerization in early stage Alzheimer’s disease brain. Acta Neuropathol Commun.

[20] Fields CR, Bengoa-Vergniory N, Wade-Martins R (2019). Targeting alpha-synuclein as a therapy for Parkinson’s disease. Front Mol Neurosci.

[21] Fleeman RM, Proctor EA (2021). Astrocytic propagation of tau in the context of Alzheimer’s disease. Front Cell Neurosci.

[22] Giasson BI, Jakes R, Goedert M, Duda JE, Leight S, Trojanowski JQ, Lee VM (2000). A panel of epitope-specific antibodies detects protein domains distributed throughout human alpha-synuclein in Lewy bodies of Parkinson’s disease. J Neurosci Res.

[23] Goedert M, Masuda-Suzukake M, Falcon B (2017). Like prions: the propagation of aggregated tau and alpha-synuclein in neurodegeneration. Brain.

[24] Guillozet-Bongaarts AL, Garcia-Sierra F, Reynolds MR, Horowitz PM, Fu Y, Wang T, Cahill ME, Bigio EH, Berry RW, Binder LI (2005). Tau truncation during neurofibrillary tangle evolution in Alzheimer’s disease. Neurobiol Aging.

[25] Halliday GM, Stevens CH (2011). Glia: initiators and progressors of pathology in Parkinson’s disease. Mov Disord.

[26] Hyman BT, Phelps CH, Beach TG, Bigio EH, Cairns NJ, Carrillo MC, Dickson DW, Duyckaerts C, Frosch MP, Masliah E (2012). National Institute on Aging-Alzheimer’s Association guidelines for the neuropathologic assessment of Alzheimer’s disease. Alzheimers Dement.

[27] Jabbari E, Duff KE (2021). Tau-targeting antibody therapies: too late, wrong epitope or wrong target?. Nat Med.

[28] Jack CR (2022). Advances in Alzheimer’s disease research over the past two decades. Lancet Neurol.

[29] Jack CR, Bennett DA, Blennow K, Carrillo MC, Dunn B, Haeberlein SB, Holtzman DM, Jagust W, Jessen F, Karlawish J (2018). NIA-AA Research Framework: toward a biological definition of Alzheimer’s disease. Alzheimers Dement.

[30] Jadhav S, Avila J, Scholl M, Kovacs GG, Kovari E, Skrabana R, Evans LD, Kontsekova E, Malawska B, de Silva R (2019). A walk through tau therapeutic strategies. Acta Neuropathol Commun.

[31] Kampers T, Friedhoff P, Biernat J, Mandelkow EM, Mandelkow E (1996). RNA stimulates aggregation of microtubule-associated protein tau into Alzheimer-like paired helical filaments. FEBS Lett.

[32] Kan A, Mohamedali A, Tan SH, Cheruku HR, Slapetova I, Lee LY, Baker MS (2013). An improved method for the detection and enrichment of low-abundant membrane and lipid raft-residing proteins. J Proteomics.

[33] Kovacs GG (2020). Astroglia and tau: new perspectives. Front Aging Neurosci.

[34] Kovacs GG, Ferrer I, Grinberg LT, Alafuzoff I, Attems J, Budka H, Cairns NJ, Crary JF, Duyckaerts C, Ghetti B (2016). Aging-related tau astrogliopathy (ARTAG): harmonized evaluation strategy. Acta Neuropathol.

[35] Kraybill ML, Larson EB, Tsuang DW, Teri L, McCormick WC, Bowen JD, Kukull WA, Leverenz JB, Cherrier MM (2005). Cognitive differences in dementia patients with autopsy-verified AD, Lewy body pathology, or both. Neurology.

[36] Kumar ST, Jagannath S, Francois C, Vanderstichele H, Stoops E, Lashuel HA (2020). How specific are the conformation-specific α-synuclein antibodies? Characterization and validation of 16 α-synuclein conformation-specific antibodies using well-characterized preparations of α-synuclein monomers, fibrils and oligomers with distinct structures and morphology. Neurobiol Dis.

[37] Lang AE, Siderowf AD, Macklin EA, Poewe W, Brooks DJ, Fernandez HH, Rascol O, Giladi N, Stocchi F, Tanner CM (2022). Trial of cinpanemab in early Parkinson’s disease. N Engl J Med.

[38] Li L, Shi R, Gu J, Tung YC, Zhou Y, Zhou D, Wu R, Chu D, Jin N, Deng K (2021). Alzheimer’s disease brain contains tau fractions with differential prion-like activities. Acta Neuropathol Commun.

[39] Luna-Munoz J, Chavez-Macias L, Garcia-Sierra F, Mena R (2007). Earliest stages of tau conformational changes are related to the appearance of a sequence of specific phospho-dependent tau epitopes in Alzheimer’s disease. J Alzheimers Dis.

[40] Malek-Ahmadi M, Beach TG, Zamrini E, Adler CH, Sabbagh MN, Shill HA, Jacobson SA, Belden CM, Caselli RJ, Woodruff BK (2019). Faster cognitive decline in dementia due to Alzheimer disease with clinically undiagnosed Lewy body disease. PLoS ONE.

[41] Mate de Gerando A, d’Orange M, Augustin E, Josephine C, Auregan G, Gaudin-Guerif M, Guillermier M, Herard AS, Stimmer L, Petit F (2021). Neuronal tau species transfer to astrocytes and induce their loss according to tau aggregation state. Brain.

[42] Matsunaga S, Fujishiro H, Takechi H (2019). Efficacy and safety of glycogen synthase kinase 3 inhibitors for Alzheimer’s disease: a systematic review and meta-analysis. J Alzheimers Dis.

[43] Mazanetz MP, Fischer PM (2007). Untangling tau hyperphosphorylation in drug design for neurodegenerative diseases. Nat Rev Drug Discov.

[44] McFarthing K, Rafaloff G, Baptista M, Mursaleen L, Fuest R, Wyse RK, Stott SRW (2022). Parkinson’s disease drug therapies in the clinical trial pipeline: 2022 update. J Parkinsons Dis.

[45] McKhann GM, Knopman DS, Chertkow H, Hyman BT, Jack CR, Kawas CH, Klunk WE, Koroshetz WJ, Manly JJ, Mayeux R (2011). The diagnosis of dementia due to Alzheimer’s disease: recommendations from the National Institute on Aging-Alzheimer’s Association workgroups on diagnostic guidelines for Alzheimer’s disease. Alzheimers Dement.

[46] Mirra SS, Heyman A, McKeel D, Sumi SM, Crain BJ, Brownlee LM, Vogel FS, Hughes JP, van Belle G, Berg L (1991). The Consortium to Establish a Registry for Alzheimer’s Disease (CERAD). Part II. Standardization of the neuropathologic assessment of Alzheimer’s disease. Neurology.

[47] Moors TE, Maat CA, Niedieker D, Mona D, Petersen D, Timmermans-Huisman E, Kole J, El-Mashtoly SF, Spycher L, Zago W (2021). The subcellular arrangement of alpha-synuclein proteoforms in the Parkinson’s disease brain as revealed by multicolor STED microscopy. Acta Neuropathol.

[48] Mukherjee S, Sakunthala A, Gadhe L, Poudyal M, Sawner AS, Kadu P, Maji SK (2023). Liquid-liquid phase separation of alpha-synuclein: a new mechanistic insight for alpha-synuclein aggregation associated with Parkinson’s disease pathogenesis. J Mol Biol.

[49] Neddens J, Temmel M, Flunkert S, Kerschbaumer B, Hoeller C, Loeffler T, Niederkofler V, Daum G, Attems J, Hutter-Paier B (2018). Phosphorylation of different tau sites during progression of Alzheimer’s disease. Acta Neuropathol Commun.

[50] Nelson PT, Alafuzoff I, Bigio EH, Bouras C, Braak H, Cairns NJ, Castellani RJ, Crain BJ, Davies P, Del Tredici K (2012). Correlation of Alzheimer disease neuropathologic changes with cognitive status: a review of the literature. J Neuropathol Exp Neurol.

[51] Oueslati A (2016). Implication of alpha-synuclein phosphorylation at S129 in synucleinopathies: what have we learned in the last decade?. J Parkinsons Dis.

[52] Pan L, Li C, Meng L, Tian Y, He M, Yuan X, Zhang G, Zhang Z, Xiong J, Chen G, Zhang Z (2022). Tau accelerates alpha-synuclein aggregation and spreading in Parkinson’s disease. Brain.

[53] Popescu A, Lippa CF, Lee VM, Trojanowski JQ (2004). Lewy bodies in the amygdala: increase of alpha-synuclein aggregates in neurodegenerative diseases with tau-based inclusions. Arch Neurol.

[54] Postuma RB, Berg D, Stern M, Poewe W, Olanow CW, Oertel W, Obeso J, Marek K, Litvan I, Lang AE (2015). MDS clinical diagnostic criteria for Parkinson’s disease. Mov Disord.

[55] Ray S, Singh N, Kumar R, Patel K, Pandey S, Datta D, Mahato J, Panigrahi R, Navalkar A, Mehra S (2020). alpha-Synuclein aggregation nucleates through liquid-liquid phase separation. Nat Chem.

[56] Rostami J, Holmqvist S, Lindstrom V, Sigvardson J, Westermark GT, Ingelsson M, Bergstrom J, Roybon L, Erlandsson A (2017). Human astrocytes transfer aggregated alpha-synuclein via tunneling nanotubes. J Neurosci.

[57] Sasaki A, Arawaka S, Sato H, Kato T (2015). Sensitive western blotting for detection of endogenous Ser129-phosphorylated alpha-synuclein in intracellular and extracellular spaces. Sci Rep.

[58] Sengupta U, Kayed R (2022). Amyloid beta, Tau, and alpha-Synuclein aggregates in the pathogenesis, prognosis, and therapeutics for neurodegenerative diseases. Prog Neurobiol.

[59] Shahmoradian SH, Lewis AJ, Genoud C, Hench J, Moors TE, Navarro PP, Castano-Diez D, Schweighauser G, Graff-Meyer A, Goldie KN (2019). Lewy pathology in Parkinson’s disease consists of crowded organelles and lipid membranes. Nat Neurosci.

[60] Siegert A, Rankovic M, Favretto F, Ukmar-Godec T, Strohaker T, Becker S, Zweckstetter M (2021). Interplay between tau and alpha-synuclein liquid-liquid phase separation. Protein Sci.

[61] Sorrentino ZA, Giasson BI (2020). The emerging role of α-synuclein truncation in aggregation and disease. J Biol Chem.

[62] Sorrentino ZA, Giasson BI, Chakrabarty P (2019). alpha-Synuclein and astrocytes: tracing the pathways from homeostasis to neurodegeneration in Lewy body disease. Acta Neuropathol.

[63] Sorrentino ZA, Goodwin MS, Riffe CJ, Dhillon JS, Xia Y, Gorion KM, Vijayaraghavan N, McFarland KN, Golbe LI, Yachnis AT, Giasson BI (2019). Unique alpha-synuclein pathology within the amygdala in Lewy body dementia: implications for disease initiation and progression. Acta Neuropathol Commun.

[64] Spina S, La Joie R, Petersen C, Nolan AL, Cuevas D, Cosme C, Hepker M, Hwang JH, Miller ZA, Huang EJ (2021). Comorbid neuropathological diagnoses in early versus late-onset Alzheimer’s disease. Brain.

[65] Thal DR, Griffin WS, de Vos RA, Ghebremedhin E (2008). Cerebral amyloid angiopathy and its relationship to Alzheimer’s disease. Acta Neuropathol.

[66] Thal DR, Rub U, Orantes M, Braak H (2002). Phases of A beta-deposition in the human brain and its relevance for the development of AD. Neurology.

[67] Twohig D, Nielsen HM (2019). alpha-Synuclein in the pathophysiology of Alzheimer’s disease. Mol Neurodegener.

[68] Walker L, McAleese KE, Thomas AJ, Johnson M, Martin-Ruiz C, Parker C, Colloby SJ, Jellinger K, Attems J (2015). Neuropathologically mixed Alzheimer’s and Lewy body disease: burden of pathological protein aggregates differs between clinical phenotypes. Acta Neuropathol.

[69] Wegmann S, Eftekharzadeh B, Tepper K, Zoltowska KM, Bennett RE, Dujardin S, Laskowski PR, MacKenzie D, Kamath T, Commins C (2018). Tau protein liquid-liquid phase separation can initiate tau aggregation. EMBO J.

[70] Wesseling H, Mair W, Kumar M, Schlaffner CN, Tang S, Beerepoot P, Fatou B, Guise AJ, Cheng L, Takeda S (2020). Tau PTM profiles identify patient heterogeneity and stages of Alzheimer’s disease. Cell.

[71] Zetterberg H, Bendlin BB (2021). Biomarkers for Alzheimer’s disease-preparing for a new era of disease-modifying therapies. Mol Psychiatry.

[72] Zhang J, Li X, Li JD (2019). The roles of post-translational modifications on alpha-synuclein in the pathogenesis of Parkinson’s diseases. Front Neurosci.

[73] Zhang S, Zhu R, Pan B, Xu H, Olufemi MF, Gathagan RJ, Li Y, Zhang L, Zhang J, Xiang W (2023). Post-translational modifications of soluble alpha-synuclein regulate the amplification of pathological alpha-synuclein. Nat Neurosci.

[74] Zhang X, Gao F, Wang D, Li C, Fu Y, He W, Zhang J (2018). Tau pathology in Parkinson’s disease. Front Neurol.

[75] Zhou Y, Shi J, Chu D, Hu W, Guan Z, Gong CX, Iqbal K, Liu F (2018). Relevance of phosphorylation and truncation of tau to the etiopathogenesis of Alzheimer’s disease. Front Aging Neurosci.

