# Supplementary material for: Distinct tau and alpha-synuclein molecular signatures in Alzheimer’s disease with and without Lewy bodies and Parkinson’s disease with dementia
Source: Acta Neuropathol. 2024 Jan 10;147(1):14. doi: 10.1007/s00401-023-02657-y (PMC10781859; doi:10.1007/s00401-023-02657-y)
Supplement: Supplementary file 1 — Supplementary file1 (DOCX 7808 KB) [file 401_2023_2657_MOESM1_ESM.docx]

**Supplementary file**

Distinct tau and alpha-synuclein molecular signatures in Alzheimer’s disease with and without Lewy bodies and Parkinson’s disease with dementia

*Bram L. van der Gaag^1,2^, Natasja A.C. Deshayes^1^, John J.P. Breve^1^, John G.J.M. Bol^1^, Allert J. Jonker^1^, Jeroen J.M. Hoozemans^2,3^, Jean-Philippe Courade^4^, Wilma D.J. van de Berg^1,2^*

^1^Amsterdam UMC, Vrije Universiteit, department of Anatomy and Neurosciences, section Clinical Neuroanatomy and Biobanking, Amsterdam, The Netherlands

^2^Amsterdam Neuroscience, program Neurodegeneration, Amsterdam, The Netherlands

^3^Amsterdam UMC, Vrije Universiteit, department of Pathology, Amsterdam, The Netherlands

^4^Discoveric Bio alpha Ltd., Pfäffikon, Schwyz, Switzerland

Corresponding author:

Name: Wilma D.J. van de Berg, PhD

E-mail: wdj.vandeberg@amsterdamumc.nl

Telephone: +31-618239398

**Supplementary methods**

**Quantification of blotting**

For semi-quantitative analyses of both the dot blots and western blots, Image Studio Software (version 5.2) was used. Measurement areas were determined for quantification; either dots for the dot blots or bands for the western blots. Definitions for the parameters used by the software were as follows:

- **Total:** sum of the individual pixel intensities in an enclosed shape.
- **Bkgnd:** value designated for background subtraction. In most cases, this is the average or median pixel intensity of pixels in the region chosen as background.
- **Area:** Total number of pixels enclosed by a shape.
- **Signal**: sum of the pixel intensity values (total) for a shape minus the product of the Bkgnd and the Area. Signal = Total – (Bkgnd x Area). The Bkgnd is multiplied by the Area to correct the Bkgnd value for the size of the shape being quantified, so the size of the shape does not need to be the same for each feature in an image.

For the background determination, the median pixel intensity of 3 pixels width around the measurement area was taken. To determine the relative amount of protein of interest to the total protein loaded (termed normalized value; NV), we used both the signal that was detected by the antibody (as measured in the 800 channel) and the signal which was detected by the Revert total protein stain (as measured in the 700 channel). This lead to the following formula for quantification of the dot-blots, which was adapted and modified from an earlier publication[1]:

$${Normalized Value}_{800}= \frac{{Signal}_{800}}{{Signal}_{Revert700}}$$

Western blot membranes however were multiplexed, and therefore multiple variants of tau or aSyn were detected on the same membrane. To eliminate any potential artefacts which might arise from application of the total protein stain, we corrected the detected signal in the 700 and 800 channels by subtracting any remaining signal after total protein removal in the region of interest where we also measured the protein of interest. This lead to formulation of the following formulas[1]:

$${Normalized Value}_{700}=\frac{({Signal}_{700}-{Signal}_{Destain700})}{{Signal}_{Revert700}}$$

$${Normalized Value}_{800}=\frac{({Signal}_{800}-{Signal}_{Destain800})}{{Signal}_{Revert700}}$$

If the calculated normalized value were a negative value, indicating that the background signal was higher that the detected signal for the protein of interest, the normalized value was returned to zero (if NV < 0, then NV = 0).

**Immunohistochemistry and scoring of p-tau and p-aSyn pathology on cryosections from frozen tissue blocks used for biochemical experiments**

To evaluate the presence and load of pathological tau and aSyn in the frozen tissue blocks, 10 µm thin cryosections were stained for pS202/T205 Tau (clone AT8, ThermoFisher, cat# MN1020, 1:1000 dilution) and pS129 aSyn (clone EP1536Y, Abcam, cat# ab51253, 1:4000 dilution) using immunohistochemistry. Sections were fixed for 20 min using 4% formaldehyde solution at room temperature (RT). Endogenous peroxidase activity in the tissue was blocked with 1% H2O2 for 30 min. Nonspecific binding was blocked by incubating the sections in 3% normal donkey serum (NDS) in TBS for 30 min. Primary antibody reaction took place for 1 h at RT and sections were stained with secondary detection solutions Envision anti-mouse/anti-rabbit (DAKO CAT# K400111-2/K400311-2) for 30 min at RT. Color was developed using the chromogen 3,3’-diaminobenzidine (DAB) and the reaction took place for 10 min (EP1536Y) or 12 min (AT8). Nuclear counterstaining was performed in hematoxylin for 20 s, after which sections were washed under running tap water for 5 min. Sequential dehydration series followed in order: 1 x 2 min 70%, 1 x 2 min 80%, 2 x 2 min 96%, 2 x 2 min 100% EtOH and 3 x 2 min xylene. Entellan (Merck, cat# 107960) was used as mounting medium when cover-slipping. After cover-slipping, sections were left to dry overnight in the fume hood. Mean scores were calculated based on the scores of two independent blinded scorers, which ranged from 0-3 for both tau and aSyn pathological features. For p-tau, the following definitions were used in 100x microscopic field of view: 0, 0 NFTs; 1, <10 NFTs; 2, 10-20 NFTs; 3, >20 NFTs. For aSyn, the following definitions were used: 0, no LBs or LNs in tissue section; 1, >1 LBs and sparse LNs in 200x microscopic field of interest; 2, >3 LBs and scattered LNs in 200x microscopic field of interest; 3, numerous LBs and LNs in 200x microscopic field of interest.

**Supplementary table 1: Clinicopathological details of all cases.**

F, female; m, male; age in years; NFT, neurofibrillary tangles; LB, Lewy bodies; PMD, post mortem delay; AD, Alzheimer’s disease; AD-LB, Alzheimer’s Disease with Lewy bodies; PDD, Parkinson’s disease with dementia; NA, not applicable; -, not available; ARTAG, age–related tau astrogliopathy; TC, temporal cortex; AMY, amygdala; +, slight; ++, moderate; +++, severe; CVA, cerebrovascular accident; CAA, cerebral amyloid angiopathy type, APOE, apolipoprotein E variant

| **Case #** | **Group** | **Gender** | **Onset age** | **Disease duration** | **Age at death** | **Cause of death** | **PMD (h)** | **Brain weight (g)** | **Braak NFT stage** | **Braak LB stage** | **Thal phase** | **ABC**  **Score** | **CAA-Type** | **ARTAG severity in TC and AMY** | ***APOE*** |
| --- | --- | --- | --- | --- | --- | --- | --- | --- | --- | --- | --- | --- | --- | --- | --- |
| 1 | Control | F | NA | NA | 71 | lung carcinoma | 6:50 | 1165 | I | 0 | 2 | A1B1C0 | - | TC: - AMY: - | - |
| 2 | Control | F | NA | NA | 77 | unknown | 4:35 | 1090 | II | 0 | 2 | A1B1C0 | - | TC: - AMY: - | - |
| 3 | Control | M | NA | NA | 85 | euthanasia | 9:22 | 1435 | I | 0 | 1 | A1B1C0 | - | TC: no AMY: ++ | - |
| 4 | Control | M | NA | NA | 68 | euthanasia | 8:40 | 1322 | I | 0 | 2 | A1B1C0 | - | TC: - AMY: - | - |
| 5 | Control | M | NA | NA | 82 | palliative sedation | 10:39 | 1305 | I | 0 | 1 | A1B1C0 | - | TC: no AMY: no | - |
| 6 | Control | M | NA | NA | 67 | euthanasia | 8:50 | 1369 | II | 0 | 1 | A1B1C0 | - | TC: no AMY: +++ | - |
| 7 | Control | F | NA | NA | 72 | heart failure | 7:20 | 1205 | 0 | 0 | 0 | A0B0C0 | - | TC: - AMY: - | - |
| 8 | Control | M | NA | NA | 77 | pneumonia | 11:25 | 1260 | I | 0 | 1 | A1B1C0 | - | TC: - AMY: - | - |
| 9 | Control | F | NA | NA | 76 | euthanasia | 7:50 | 1098 | I | 0 | 2 | A1B1C0 | - | TC: no AMY: no | - |
| 10 | Control | F | NA | NA | 78 | unknown | 10:00 | 1175 | I | 0 | 1 | A1B1C0 | - | TC: no AMY: - | - |
| 11 | AD | M | 79 | 7 | 86 | palliative sedation | 3:45 | 1406 | IV | 0 | 5 | A3B2C2 | 1 | TC: + AMY: + | - |
| 12 | AD | F | 78 | 10 | 88 | CVA | 6:40 | 905 | V | 0 | 5 | A3B3C3 | 1 | TC: + AMY: ++ | 22 |
| 13 | AD | F | 64 | 12 | 76 | euthanasia | 3:45 | 1155 | V | 0 | 5 | A3B3C3 | 1 | TC: no AMY: no | - |
| 14 | AD | F | 82 | 3 | 85 | heart failure | 7:20 | 1317 | VI | 0 | 4 | A3B3C3 | 1 | TC: - AMY: +++ | - |
| 15 | AD | M | 57 | 12 | 69 | respiratory failure | 11:55 | - | V | 0 | 5 | A3B3C3 | 1 | TC: no AMY: + | - |
| 16 | AD | M | 58 | 10 | 68 | euthanasia | 9:15 | - | V | 0 | 5 | A3B3C3 | 2 | TC: no AMY: no | - |
| 17 | AD | M | 70 | 14 | 84 | euthanasia | 8:35 | - | IV | 0 | 3 | A2B2C2 | - | TC: + AMY: ++ | - |
| 18 | AD | M | 66 | 11 | 77 | septic cardiogenic shock | 9:05 | - | VI | 0 | 5 | A3B3C3 | 1 | TC: no AMY: + | - |
| 19 | AD | M | 67 | 10 | 76 | cachexia, dehydration | 4:40 | 1300 | VI | 0 | 5 | A3B3C3 | 1 | TC: + AMY: ++ | - |
| 20 | AD | F | 81 | 9 | 90 | cardiac arrest | 9:00 | 1275 | IV | 0 | 4 | A3B2C1 | 1 | TC: +++ AMY: +++ | - |
| 21 | AD-LB | F | 80 | 10 | 90 | cardiac arrest | 6:30 | 865 | VI | VI | 5 | - | - | TC: - AMY: - | - |
| 22 | AD-LB | M | 63 | 7 | 70 | pneumonia | 5:10 | 1235 | V | VI | 4 | A3B3C3 | - | TC: - AMY: no | - |
| 23 | AD-LB | M | 71 | 15 | 86 | stopped eating and drinkining | 5:05 | 1050 | VI | VI | 5 | A3B3C3 | 1 | TC: + AMY: +++ | 43 |
| 24 | AD-LB | M | 67 | 6 | 73 | pulmonary embolism | 7:55 | 1310 | IV | VI | 3 | A2B2C2 | 1 | TC: no AMY: no | - |
| 25 | AD-LB | F | 68 | 13 | 81 | cachexia, dehydration | 4:50 | 936 | VI | IV | 5 | A3B3C3 | 1 | TC: + AMY: + | - |
| 26 | AD-LB | F | 82 | 5 | 87 | acute renal failure | 5:00 | 1060 | VI | VI | 4 | A3B3C3 | 2 | TC: no AMY: +++ | - |
| 27 | AD-LB | F | 73 | 8 | 81 | general deterioration | 4:05 | 1021 | V | VI | 5 | A3B3C3 | 2 | TC: + AMY: no | - |
| 28 | AD-LB | M | - | - | 77 | palliative sedation | 4:25 | 1391 | IV | VI | 3 | A2B2C2 | 2 | TC: + AMY: no | - |
| 29 | AD-LB | M | 69 | 6 | 75 | general deterioration | 6:15 | 1070 | V | VI | 4 | A3B3C3 | - | TC: - AMY: + | - |
| 30 | AD-LB | M | 71 | 7 | 78 | - | 5:00 | 1240 | IV | VI | 3/4 | A2B2C0 | - | TC: + AMY: - | - |
| 31 | PDD | M | 69 | 6 | 75 | euthanasia | 3:35 | 1310 | II | VI | 1 | A1B1C0 | - | TC: - AMY: + | - |
| 32 | PDD | M | 60 | 21 | 81 | euthanasia | 6:10 | 1140 | III | VI | 0 | A0B2C0 | - | TC: - AMY: +++ | - |
| 33 | PDD | F | 82 | 6 | 88 | euthanasia | 6:05 | 955 | II | VI | 1 | A1B1C0 | - | TC: - AMY: + | - |
| 34 | PDD | F | 72 | 17 | 89 | dehydration, cachexia, sysphagia | 6:35 | 1065 | II | VI | 1 | A1B1C0 | - | TC: - AMY: + | - |
| 35 | PDD | M | 53 | 7 | 60 | euthanasia | 8:30 | 1365 | 0 | VI | 0 | A0B0C0 | - | TC: - AMY: - | - |
| 36 | PDD | M | 76 | 8 | 84 | malignancy, cachexia | 4:50 | 1430 | III | V | 1 | A0B2C0 | - | TC: no AMY: +++ | - |
| 37 | PDD | M | 53 | 19 | 72 | dehydration | 3:50 | 1435 | I | VI | 1 | A1B1C0 | - | TC: - AMY: + | - |
| 38 | PDD | M | 65 | 6 | 71 | euthanasia | 4:55 | 1391 | I | VI | 3 | A2B1C0 | 2 | TC: + AMY: + | - |
| 39 | PDD | M | 63 | 17 | 80 | euthanasia | 3:50 | 131 | I | V | 1 | A1B1C0 | - | TC: - AMY: - | - |
| 40 | PDD | M | 66 | 8 | 74 | aspiration pneumonia | 8:50 | 1335 | III | VI | 2 | A1B2C0 | - | TC: - AMY: - |  |

**Supplementary table 2: Overview of selected primary antibody dilutions for blotting purposes.**

| **Target protein** | **Clone** | **Company** | **Article #** | **Host** | **Dilution** |
| --- | --- | --- | --- | --- | --- |
| aSyn | MJFR-14-6-4-2 | Abcam | ab209538 | Mouse | 1:1000 |
| aSyn | Syn-1 Clone 42 | BD Biosciences | 610786 | Mouse | 1:1000 |
| aSyn | EP1536Y | Abcam | ab51253 | Rabbit | 1:1000 |
| aSyn | LB509 | Abcam | ab27766 | Mouse | 1:1000 |
| aSyn | 5G4 | Merck Millipore | MABN389 | Mouse | 1:500 |
| aSyn | A15127A | Biolegend | 848402 | Mouse | 1:500 |
| aSyn | Clone 24.8 | Novus | NBP1-26380 | Mouse | 1:1000 |
| aSyn | ASyM Clone 4.2 | Agrisera | AS13 2719 | Mouse | 1:500 |
| aSyn | A15110D | Biolegend | 849102 | Mouse | 1:500 |
| aSyn | 4B12 | Thermo Fisher | MA1-90346 | Mouse | 1:1000 |
| aSyn | Syn204 | Abcam | ab3309 | Mouse | 1:200 |
| aSyn | MJFR1 | Abcam | ab138501 | Mouse | 1:10.000 |
| aSyn | Syn211 | Abcam | ab80627 | Mouse | 1:1000 |
| aSyn | A15115A | Biolegend | 848302 | Mouse | 1:500 |
| aSyn | 5C2 | Enzo Life Sciences | ALX-804-656-100 | Mouse | 1:1000 |
| aSyn | Syn202 | ThermoFisher | 32-8200 | Mouse | 1:500 |
| Tau | AT8 | Thermo Fisher | MN1020 | Mouse | 1:1000 |
| Tau | E178 | Abcam | ab32057 | Rabbit | 1:1000 |
| Tau | EPR2400 | Abcam | ab79540 | Rabbit | 1:1000 |
| Tau | EPR2866 | Abcam | ab79415 | Rabbit | 1:1000 |
| Tau | Tau46 | Biolegend | 806601 | Mouse | 1:500 |
| Tau | 3H6.H7 | Biolegend | 823801 | Mouse | 1:500 |
| Tau | 71C11 | Biolegend | 816801 | Mouse | 1:2000 |
| Tau | Tau-12 | Biolegend | 806501 | Mouse | 1:20.000 |
| Tau | SMI 51 | Biolegend | 836104 | Mouse | 1:500 |
| Tau | 1E1/A6 | Merck/Sigma | 05-804 | Mouse | 1:4000 |
| Tau | 1E7 | Merck/Sigma | MABN388 | Mouse | 1:1000 |
| Tau | 8E6/C11 | Merck/Sigma | 05-803 | Mouse | 1:4000 |
| Tau | Tau-1 (PC1C6) | Merck/Sigma | MAB3420 | Mouse | 1:20.000 |
| Tau | Tau C3 | Merck/Sigma | 36-017 | Mouse | 1:2000 |
| Tau | BT2 | ThermoFisher | MN1010 | Mouse | 1:500 |
| Tau | AT180 | ThermoFisher | MN1040 | Mouse | 1:200 |
| Tau | HT7 | ThermoFisher | MN1000 | Mouse | 1:1000 |
| Tau | AT100 | ThermoFisher | MN1060 | Mouse | 1:1000 |
| Tau | TAU-5 | ThermoFisher | AHB0042 | Mouse | 1:500 |
| Tau | 2H23L4 | ThermoFisher | 701054 | Rabbit | 1:1000 |

**Supplementary table 3: Multiplex primary antibody combinations for detection of different epitopes and PTMs on western blot.**

PTM, post translational modification

| **WB #** | **Target protein** | **Clone + epitope/PTM measured in 700 channel** | **Clone + epitope/PTM measured in 800 channel** |
| --- | --- | --- | --- |
| 01+02 | aSyn | Syn-1 clone 42 a.a. 91-99 | EP1536Y pS129 |
| 03+04 | aSyn | A15115A a.a. 80-96 | MJFR-14-6-4-2 a.a. 133-138 |
| 05+06 | aSyn | 4B12 a.a. 103-108 | MJFR1 a.a. 118-123 |
| 07+08 | Tau | TAU12 a.a. 8-16 | E178 pS396 |
| 09+10 | Tau | AT180 pT231 | EPR2400 pS198 |
| 11+11 | Tau | AT100 pT212/S214 | EPR2866 pS422 |

**Supplementary table 4: Overview of selected primary antibody dilutions and antigen retrieval methods for immunohistochemistry on FFPE tissue sections.**

| **Clone** | **Article #** | **Manufacturer** | **Dilution** | **Antigen retrieval method** |
| --- | --- | --- | --- | --- |
| A15115A | 848302 | BioLegend | 1/10.000 | 100% formic acid |
| AT8 | MN1020 | ThermoFisher | 1/800 | steaming in citrate buffer (pH 6.0) |
| AT100 | MN1060 | ThermoFisher | 1/400 | steaming in Tris-EDTA buffer (pH 9.0) |
| AT180 | MN1040 | ThermoFisher | 1/500 | steaming in Tris-EDTA buffer (pH 9.0) |
| E178 | ab32057 | Abcam | 1/4000 | steaming in Tris-EDTA buffer (pH 9.0) |
| EP1536Y | ab51253 | Abcam | 1/4000 | steaming in Tris-EDTA buffer (pH 9.0) |
| EPR2400 | ab79540 | Abcam | 1/4000 | steaming in Tris-EDTA buffer (pH 9.0) |
| EPR2866 | ab79415 | Abcam | 1/400 | steaming in Tris-EDTA buffer (pH 9.0) |
| MJFR1 | ab138501 | Abcam | 1/20,000 | 100% formic acid |
| MJFR14 | ab209538 | Abcam | 1/10,000 | Proteinase K in TE buffer (50mM Tris HCl, 1mM EDTA, 0.5% Triton X-100, pH 8.0) |
| SYN-1 | 610787 | BD Transduction Labs | 1/1000 | 100% formic acid |

**Supplementary results**

**Phosphorylated tau and alpha-synuclein load in selected brain regions for biochemical measures**

The presence of p-tau pathology was confirmed in the frozen MTG and AMY tissue of AD and AD-LB cases used for DB and WB. In the MTG, the pS202/T205 tau (AT8) scores were significantly higher in the AD-LB than the PDD (p = 0.04; sup. fig. S1) and controls (p = 0.04; sup. fig. S1), as well as when comparing AD to controls (p = 0.01; sup. fig. S1) and the PDD (p=0.01; sup. fig. S1) group. Higher pS202/T205 tau scores were also observed in the AMY region for the AD-LB group compared to the PDD group (p = 0.04; sup. fig. S1), while higher scores were found in the AD group compared to PDD (p = 0.001; sup. fig. S1) and controls (p < 0.01). However, no difference in pS202/T205 tau scores was observed between the AD-LB and AD groups in both regions.

The presence of pSer129 aSyn pathology was confirmed in the frozen MTG and AMY tissue of PDD and AD-LB cases, and absent in AD and controls. In the MTG, pS129 aSyn (EP1536Y) scores were significantly higher in the AD-LB group compared to the AD (p < 0.01; sup. fig. S1) and controls (p < 0.01; sup. fig. S1) groups, while no significant differences were observed between the PDD and any of the other groups (p > 0.05; sup. fig. S1). Conversely, higher pS129 aSyn scores were observed in the AMY region when comparing the AD-LB group to AD (p < 0.0001; sup. fig. S1) and control (p < 0.0001; sup. fig. S1) groups, and higher pS129 aSyn scores were also found in the PDD group compared to AD (p < 0.01; sup. fig. S1) and control (p < 0.01; sup. fig. S1) groups. Although the AD-LB group showed overall higher aSyn pathology scores compared to the PDD group, differences were non-significant (p > 0.05; sup. fig. S1).

To assess whether biochemical detection of pS202/T205 tau and pS129 aSyn reflected detected pathology as assessed by semi-quantitative scoring of frozen tissue sections from the exact same tissue blocks, correlation analyses were performed for each antibody of interest per region of interest. Strong correlations were found for normalized values as detected by DB and pathological scores for pS202/T205 tau in the MTG (r = 0.8952, p < 0.0001; sup. fig. S2-A) and AMY (r = 0.8811, p < 0.0001; sup. fig. S2-B) as well as for pS129 aSyn in the AMY (r = 0.9229, p <0.0001; sup. fig. S2-D). There was a moderate correlation for pS129 aSyn in the MTG (r= 0.5977, p < 0.0001; sup. fig. S1-C), which could be explained by the relatively low pathological aSyn scores in this region and some outliers within the dataset (no data points were removed for these analyses).

**Supplementary figure S1. Manual ordinal scoring of p-tau and pSer129 aSyn in cryosections.** pS202/T205 tau scores (AT8) were significantly higher in AD and AD-LB groups compared to PDD and control groups for both the MTG and AMY. pS129 aSyn (EP1536Y) scores were significantly higher in AD-LB compared to the AD and controls groups for both the MTG and AMY, while the PDD group only showed significantly higher p-S129 aSyn scores in the AMY when compared to the AD and controls groups. Group comparisons were performed with Kruskal-Wallis. * p < 0.05 ** p < 0.01 **** p < 0.0001

b

a


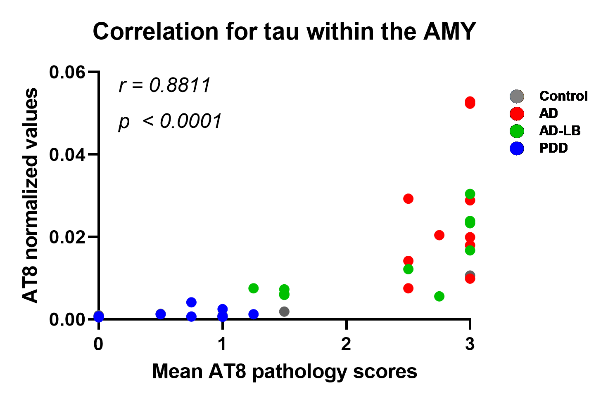


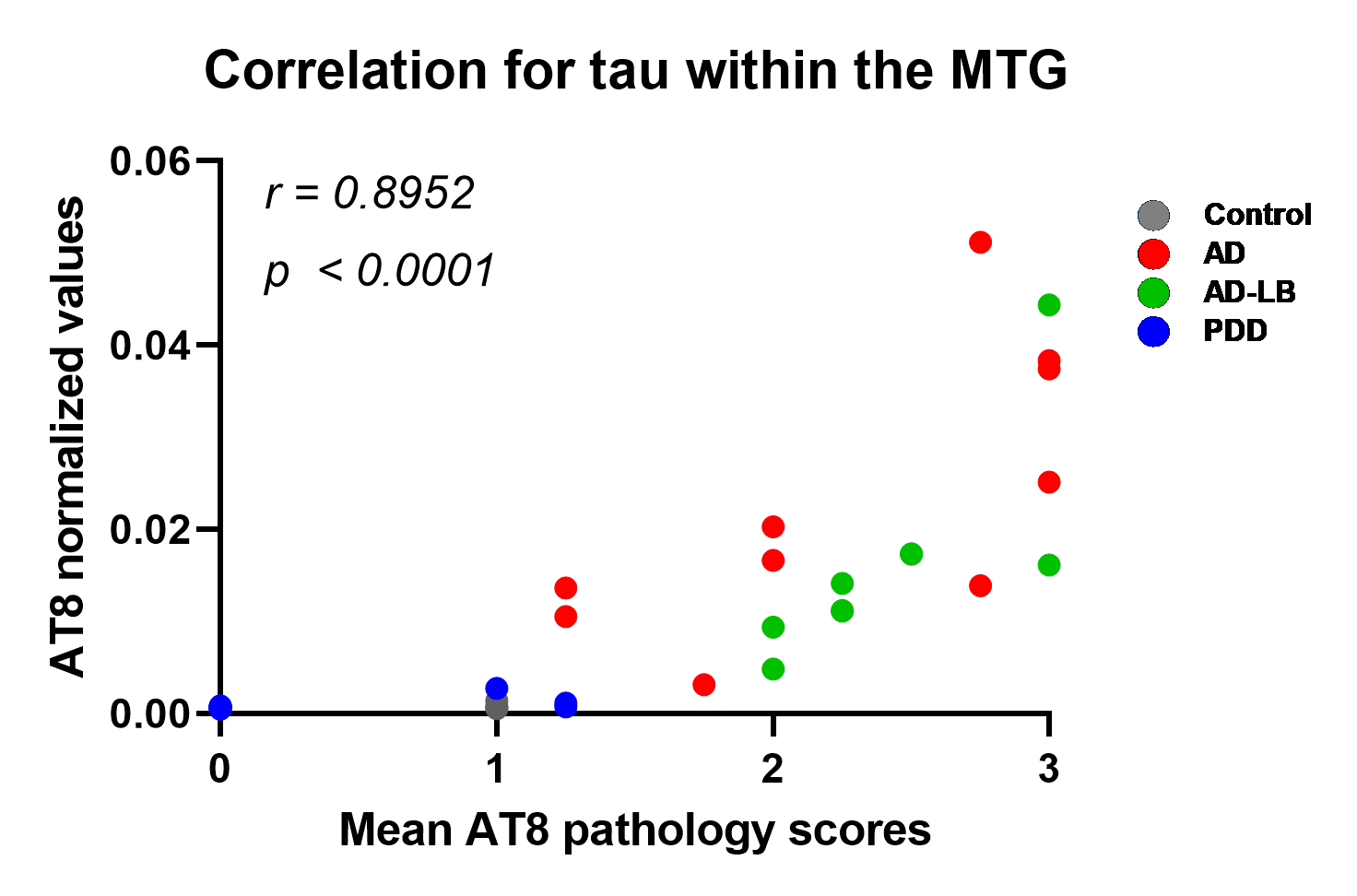


c


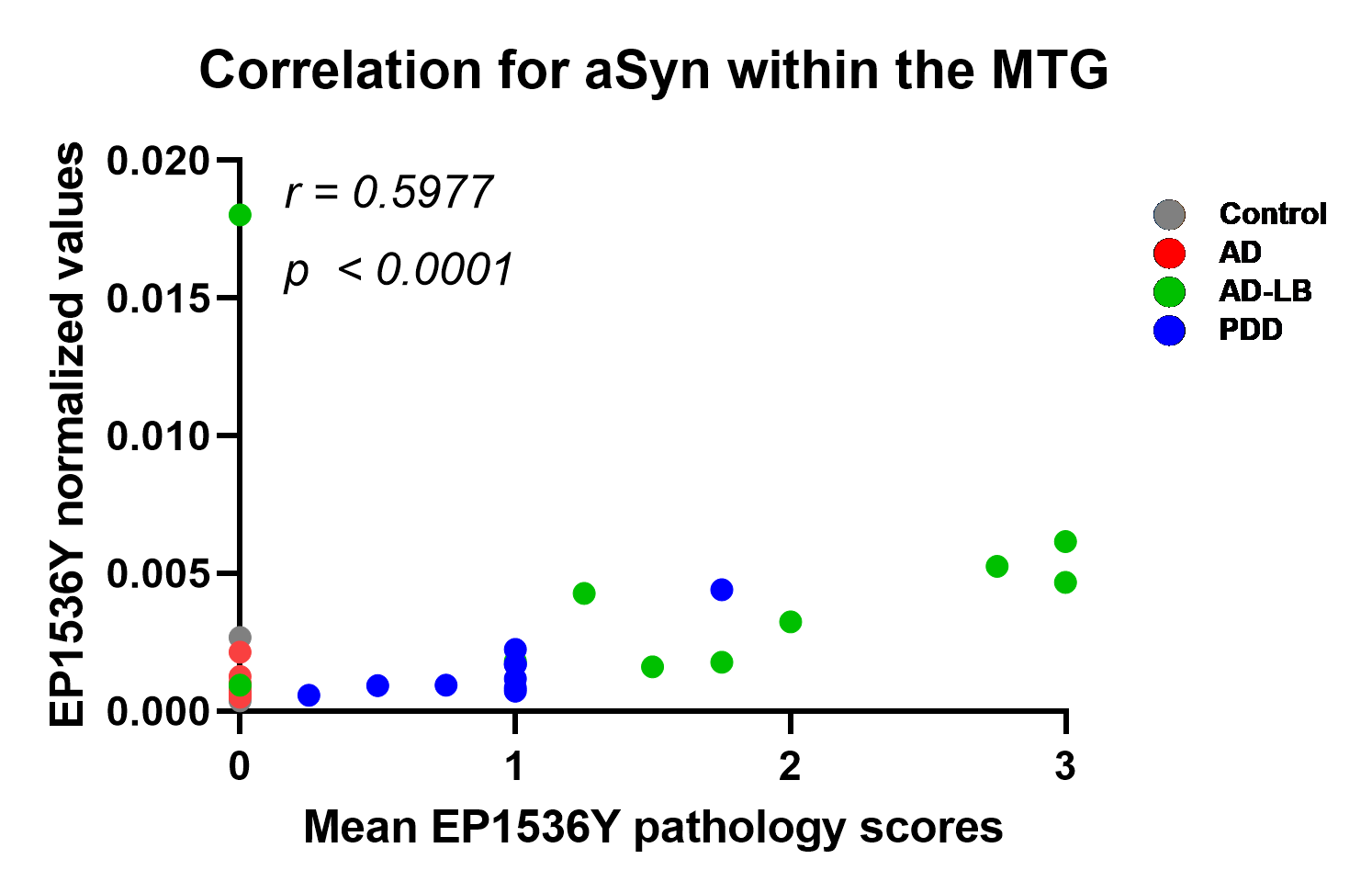

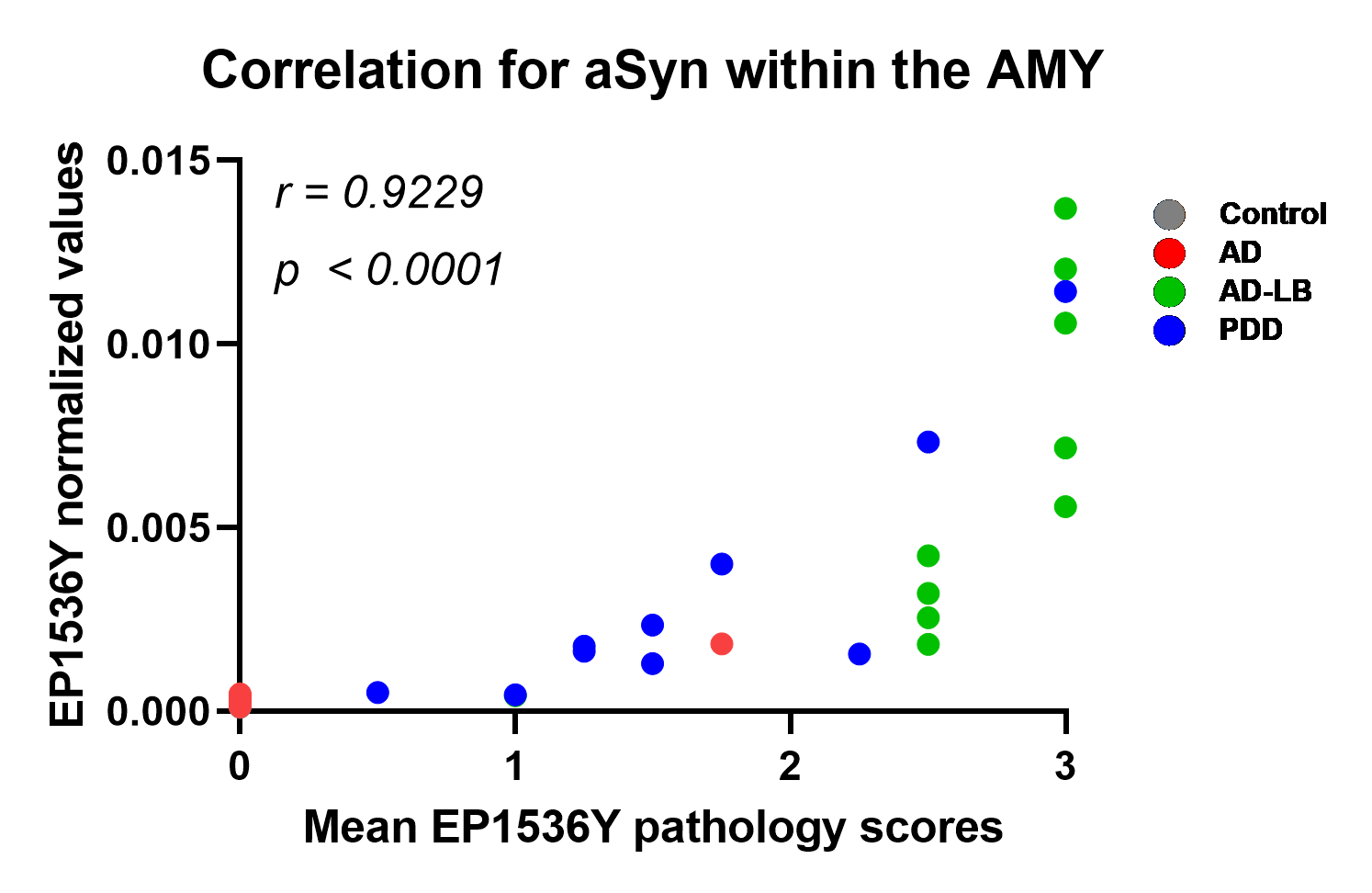


d

**Supplementary figure S2.** Correlations as determined by Spearman’s correlation analyses showing significant correlations between phospho tau (AT8) load and biochemical measurements in both the MTG (a) and AMY (b). Significant correlations were also detected for phospho aSyn (EP1536Y) load and biochemical measurements in the MTG (c) and AMY (d).

**
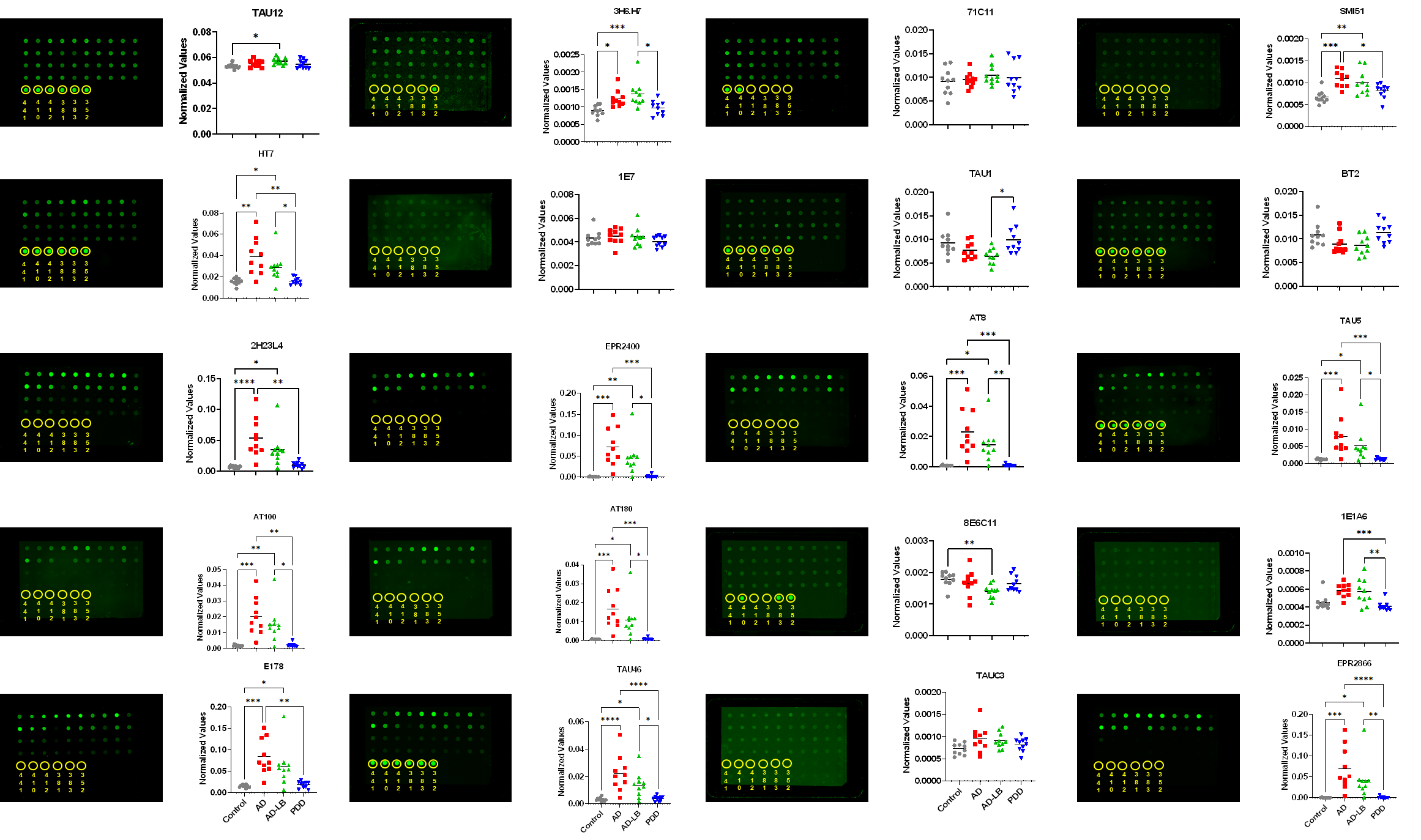
**

**Supplementary figure S3.** Individual results of dot-blots for investigated tau antibodies on the MTG. Acquisition of detected antibody is shown on the blot with results juxtaposed *(right)*. Spots loaded with recombinant tau proteins are highlighted with yellow circles, with respective amino acid length highlighted below. *, p < 0.05; **, p < 0.01; ***, p < 0.001; ****, p < 0.0001

**
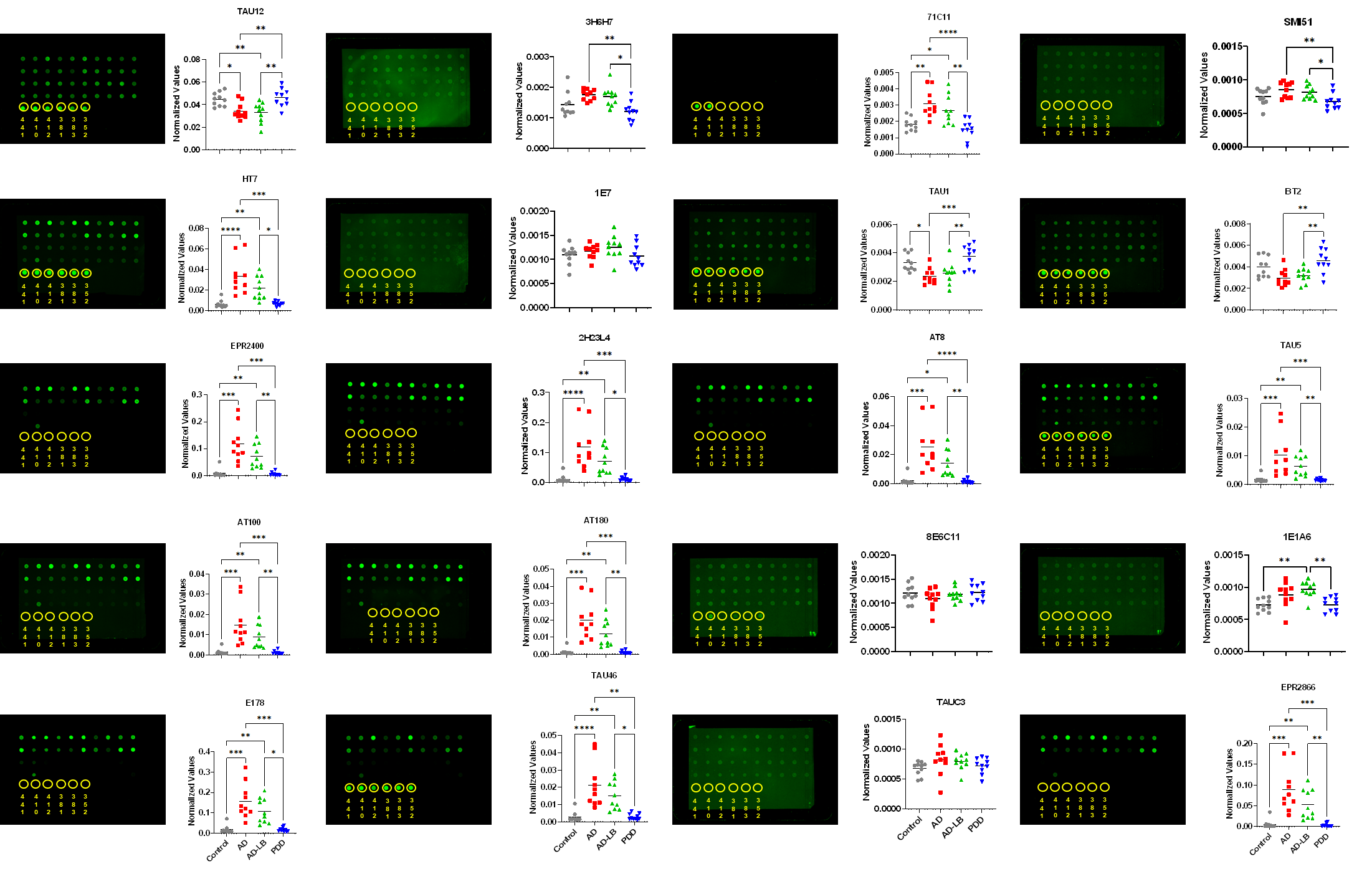
**

**Supplementary figure S4.** Individual results of dot-blots for investigated tau antibodies on the amygdala. Acquisition of detected antibody is shown on the blot with results juxtaposed *(right)*. Spots loaded with recombinant tau proteins are highlighted with yellow circles, with respective amino acid length highlighted below. *, p < 0.05; **, p < 0.01; ***, p < 0.001; ****, p < 0.0001

**
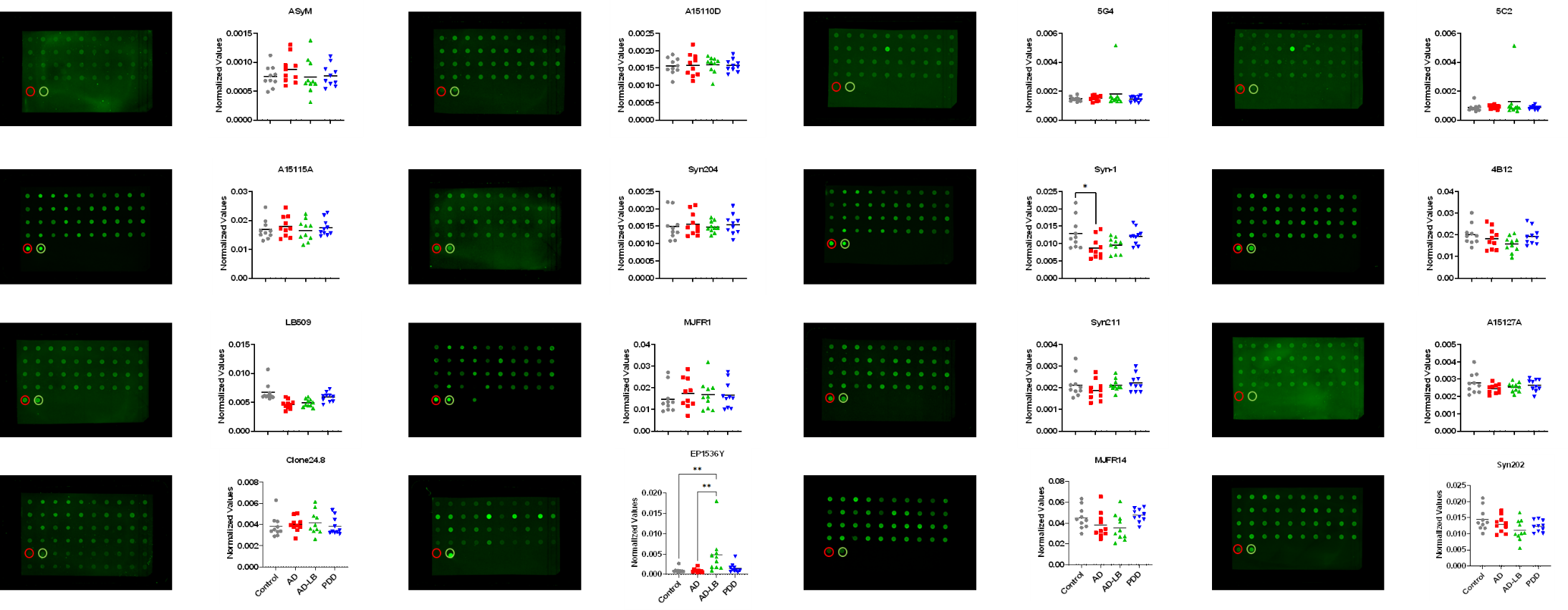
**

**Supplementary figure S5.** Individual results of dot-blots for investigated aSyn antibodies on the MTG. Acquisition of detected antibody is shown on the blot with results juxtaposed *(right)*. Spot loaded with recombinant monomeric aSyn is highlighted by a red circle, spot loaded with recombinant pS129 aSyn is highlighted by a green circle. *, p < 0.05; **, p < 0.01

**
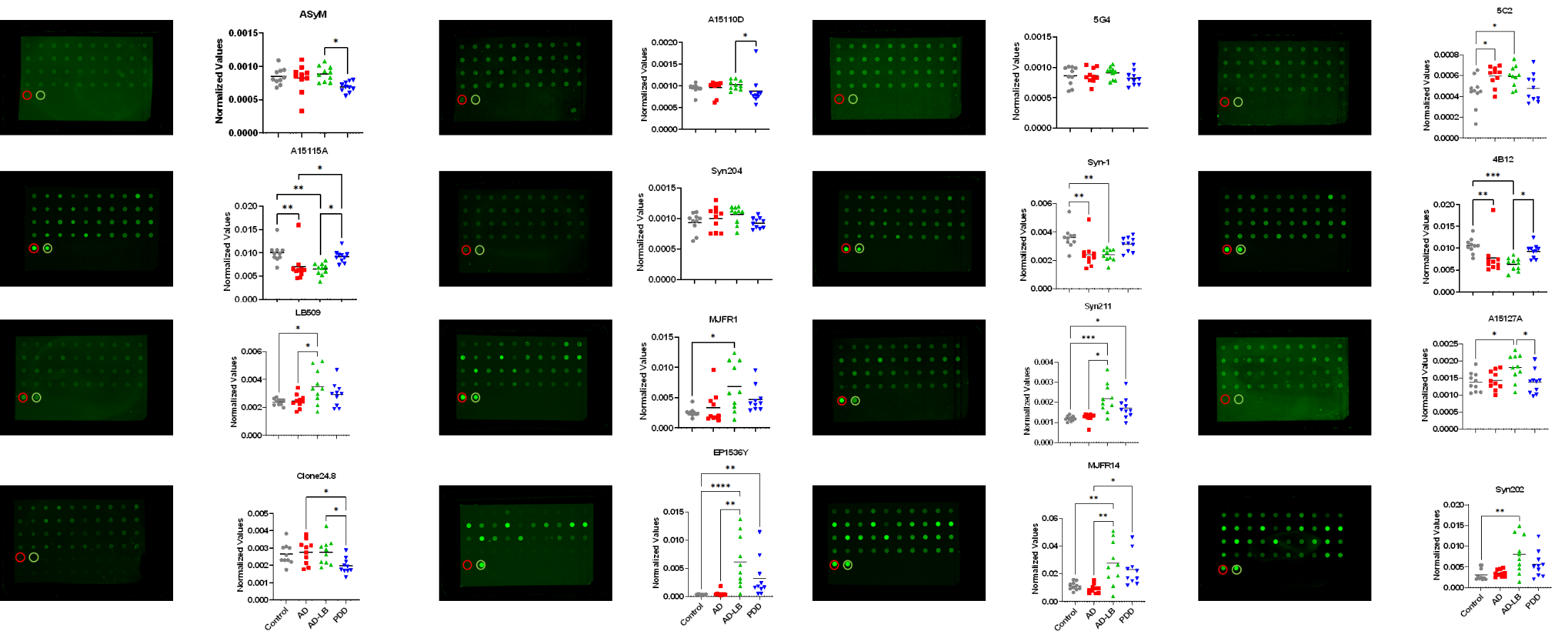
**

**Supplementary figure S6.** Individual results of dot-blots for investigated aSyn antibodies on the amygdala. Acquisition of detected antibody is shown on the blot with results juxtaposed *(right)*. Spot loaded with recombinant monomeric aSyn is highlighted by a red circle, spot loaded with recombinant pS129 aSyn is highlighted by a green circle. *, p < 0.05; **, p < 0.01; ***, p < 0.001; ****, p < 0.0001

**
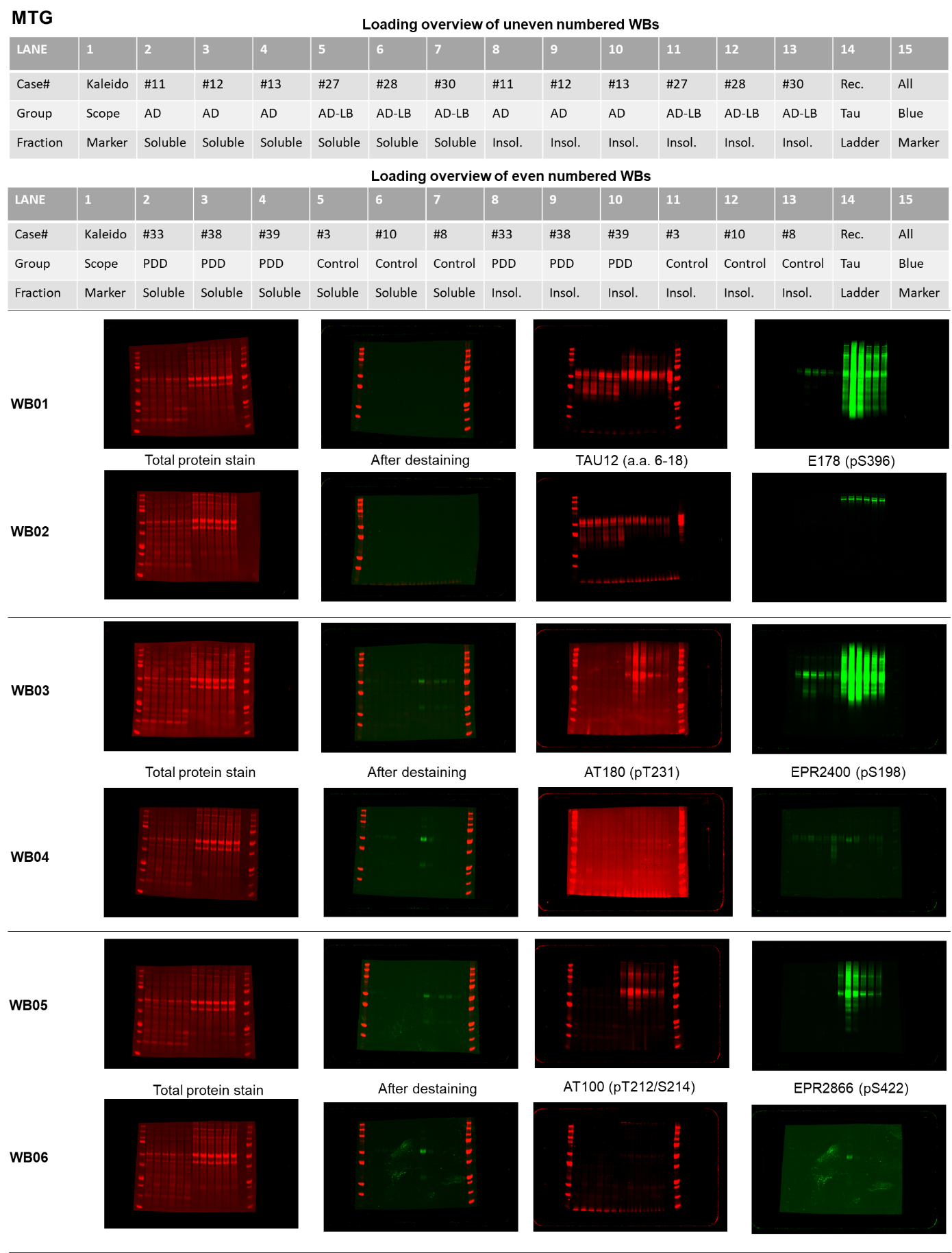
**

**Supplementary figure S7.** Full western blots for all investigated tau antibodies on MTG fractions. An overview of sample loading for all uneven (WB01-WB03-WB05) and even (WB02-WB04-WB05) numbered western blots is shown *(top)*. Acquisitions of blot after total protein stain, destaining procedure and multiplex antibody staining are shown *(bottom)*.

**
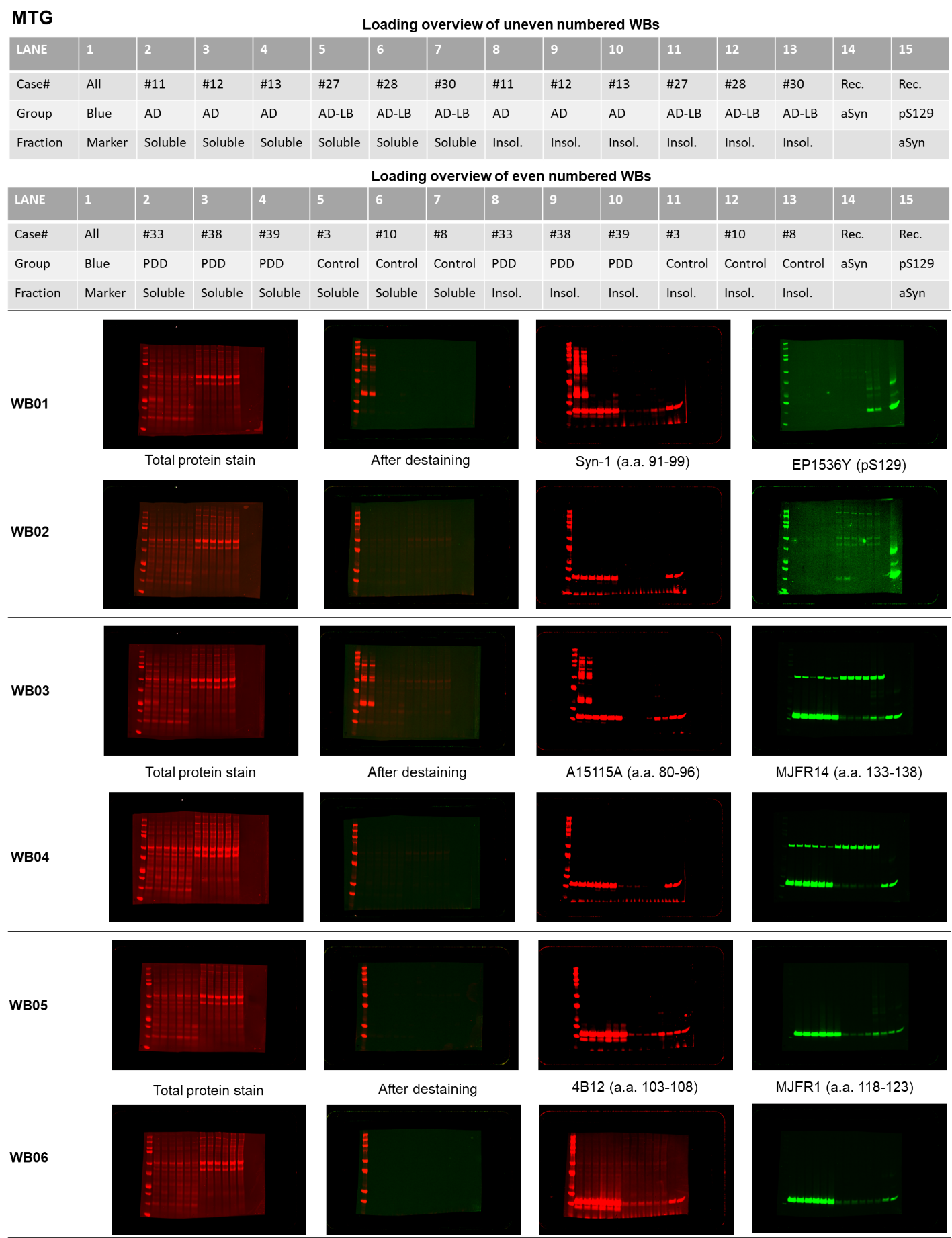
**

**Supplementary figure S8.** Full western blots for all investigated aSyn antibodies on MTG fractions. An overview of sample loading for all uneven (WB01-WB03-WB05) and even (WB02-WB04-WB05) numbered western blots is shown *(top)*. Acquisitions of blot after total protein stain, destaining procedure and multiplex antibody staining are shown *(bottom)*.

**
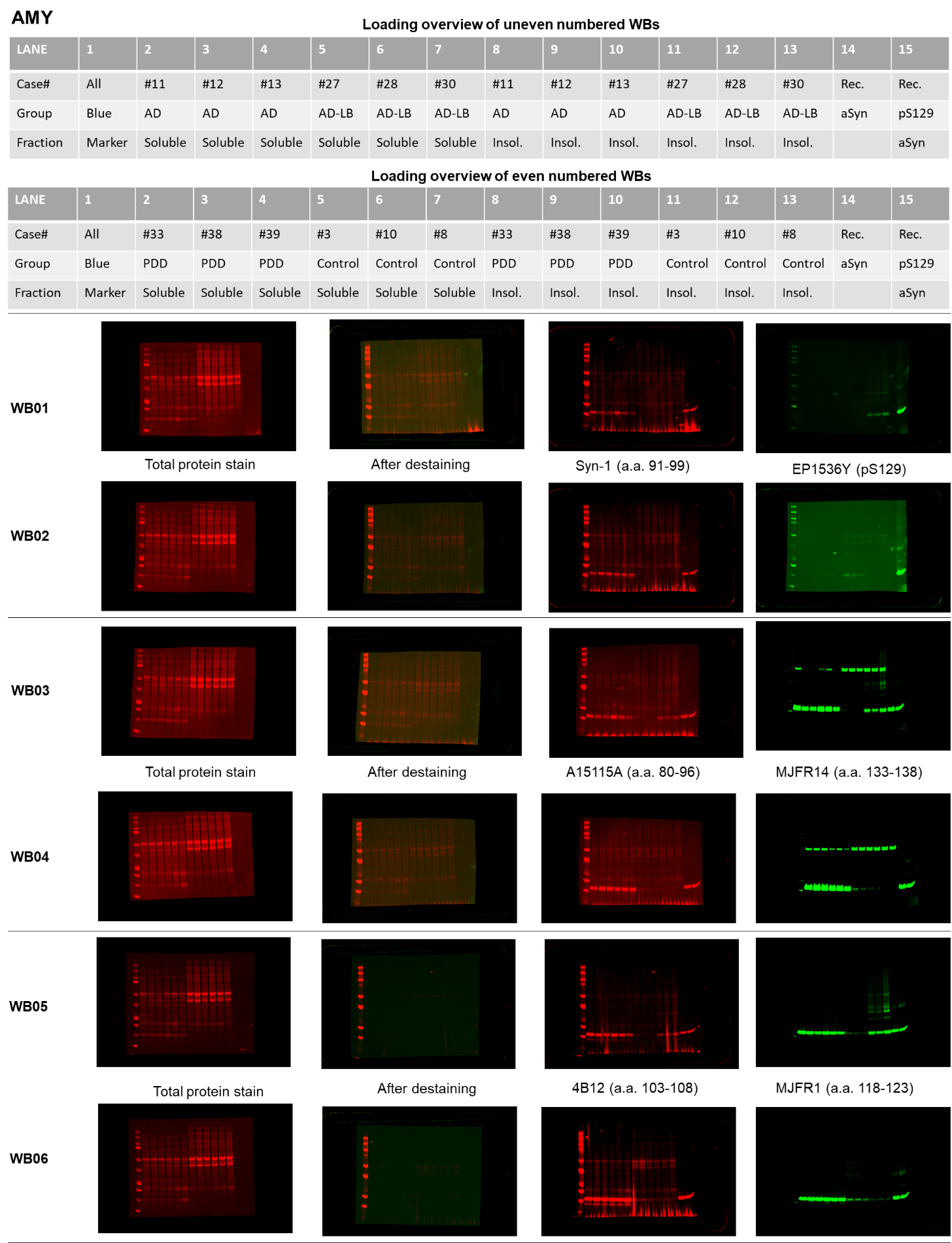
**

**Supplementary figure S9.** Full western blots for all investigated aSyn antibodies on amygdala fractions. An overview of sample loading for all uneven (WB01-WB03-WB05) and even (WB02-WB04-WB05) numbered western blots is shown *(top)*. Acquisitions of blot after total protein stain, destaining procedure and multiplex antibody staining are shown *(bottom)*.

**
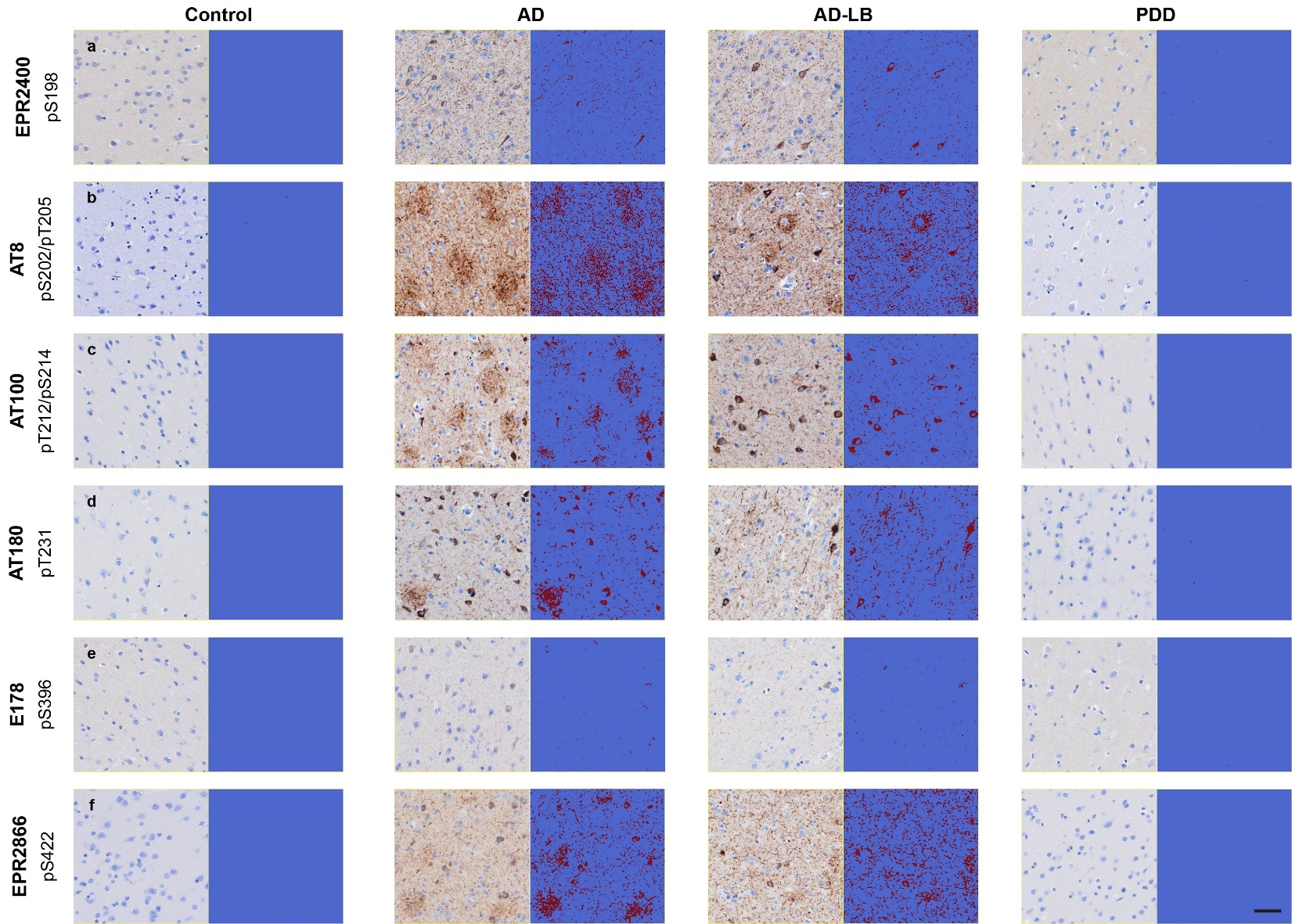
**

**Figure S10. Detection of Tau immunoreactivity by different QuPath pixel classifiers in representative immunostained MTG sections.** For the measurement of the %area immunoreactivity revealed by EPR2400 (a), AT8 (b), AT100 (c), AT180 (d), E178 (e) and EPR2866 (f), three different pixel classifiers were used in QuPath [2]. For EPR2400 and AT100 and for AT180 and EPR2866 the same pixel classifiers have been used. Shown in red is what is recognized by the specific pixel classifier as immunoreactive. Scale bar represents 50μm.

**
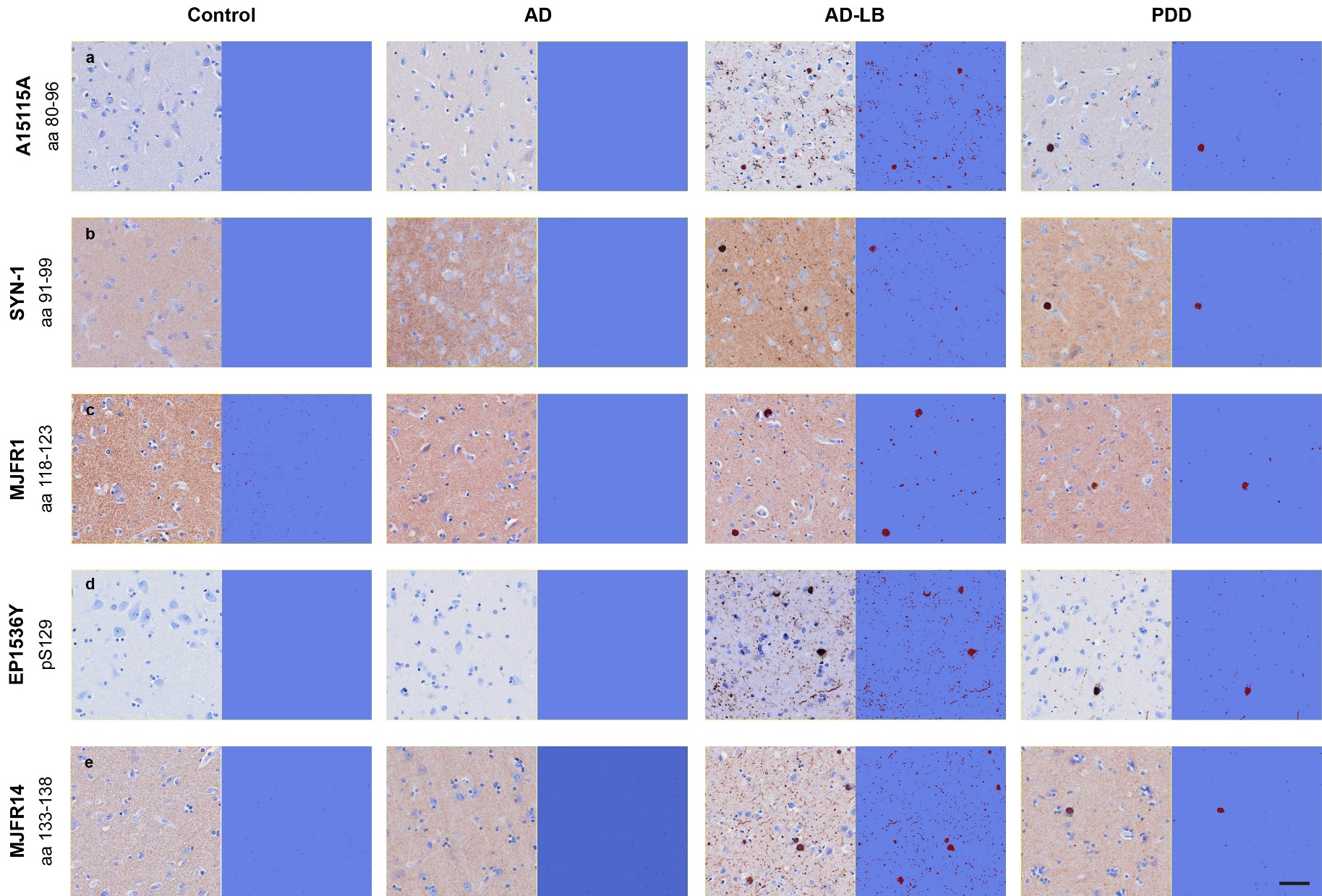
**

**Figure S11. Detection of aSyn immunoreactivity by different QuPath pixel classifiers in representative immunostained MTG sections.** For the measurement of the %area immunoreactivity revealed by A15115A (a), SYN-1 (b), MJFR1 (c), EP1536Y (d) and MJFR14 (e), five different pixel classifiers were used in QuPath [2]. Shown in red is what is recognized by the specific pixel classifier as immunoreactive. Scale bar represents 50μm.

**
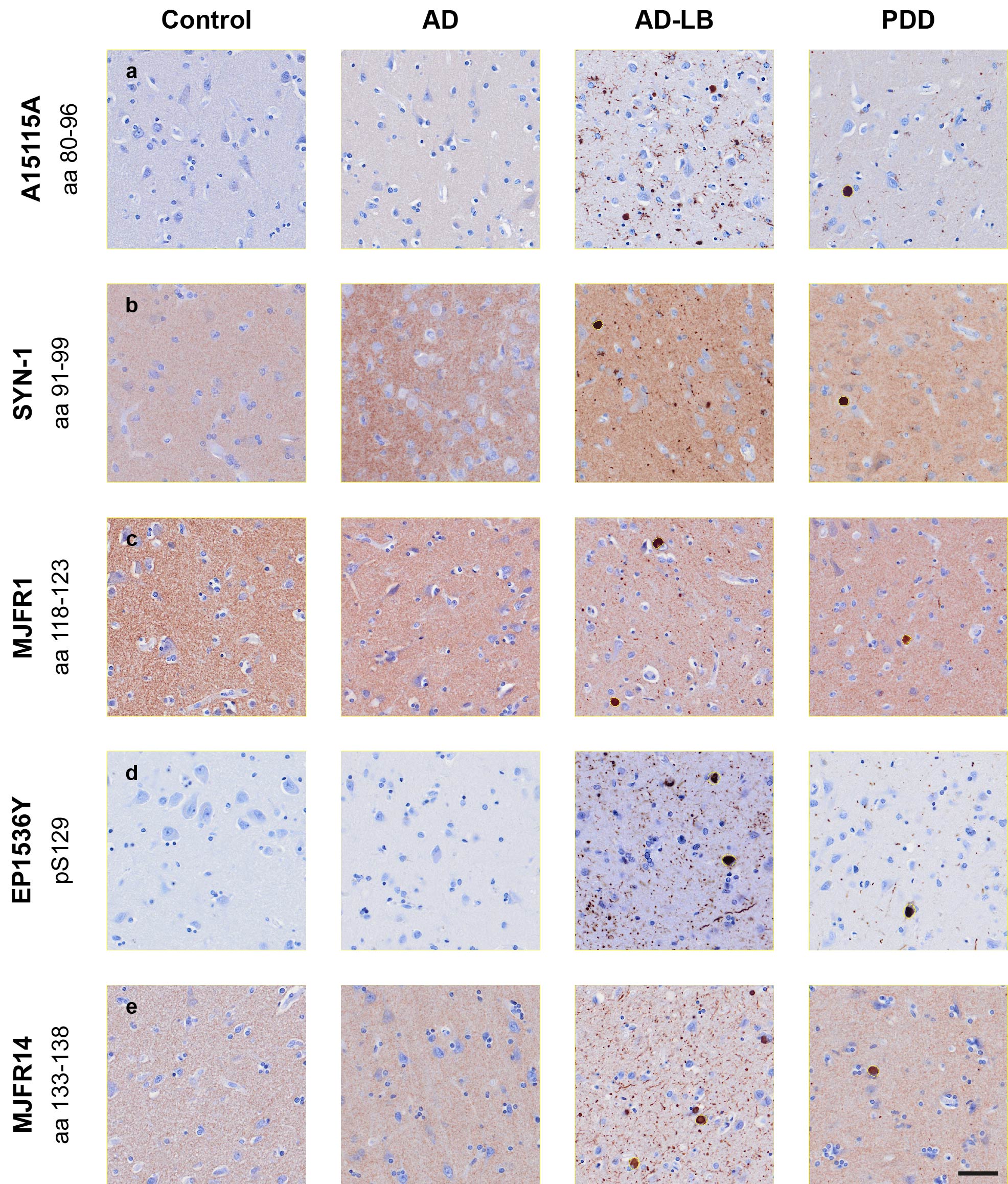
**

**Figure S12. Detection of LBs by QuPath object classifier.** An object classifier was trained in QuPath to determine LBs (5 – 25 µm in diameter) per mm^2^ for A15115A (a), SYN-1 (b), MJFR1 (c), EP1536Y (d) and MJFR14 (e) [2]. Encircled in yellow are detected LBs by the script. Scale bar represents 50 µm.

**Supplementary file references**

[1] A.C. Chlebowski, G.E. Kisby, Protocol for High-Throughput Screening of Neural Cell or Brain Tissue Protein Using a Dot-Blot Technique with Near-Infrared Imaging, STAR Protoc 1(2) (2020).

[2] P. Bankhead, M.B. Loughrey, J.A. Fernandez, Y. Dombrowski, D.G. McArt, P.D. Dunne, S. McQuaid, R.T. Gray, L.J. Murray, H.G. Coleman, J.A. James, M. Salto-Tellez, P.W. Hamilton, QuPath: Open source software for digital pathology image analysis, Sci Rep 7(1) (2017) 16878.
